# Piperlongumine conquers temozolomide chemoradiotherapy resistance to achieve immune cure in refractory glioblastoma via boosting oxidative stress-inflamation-CD8^+^-T cell immunity

**DOI:** 10.1186/s13046-023-02686-1

**Published:** 2023-05-10

**Authors:** Feng Liu, Qian Zhou, Hai-feng Jiang, Ting-ting Zhang, Cheng Miao, Xiao-hong Xu, Jia-xing Wu, Song-lin Yin, Shi-jie Xu, Jing-yi Peng, Pan-pan Gao, Xuan Cao, Feng Pan, Ximiao He, Xiao Qian Chen

**Affiliations:** 1 Department of Pathophysiology, School of Basic Medicine, Tongji Medical College, Key Laboratory of Ministry of Education for Neurological Disorders, Huazhong University of Science and Technology, Wuhan, 430030 China; 2 Department of Pharmacy, First Affiliated Hospital of Yangtze University, Jingzhou, 434000 China; 3 Department of Physiology, School of Basic Medicine, Tongji Medical College, Hubei Key Laboratory of Drug Target Research and Pharmacodynamic Evaluation, Huazhong University of Science and Technology, Wuhan, 430030 China; 4 Department of Basic Medical Science, Medical College, Taizhou University, Taizhou, 318000 China; 5 Department of Urology, Tongji Medical College, Union Hospital, Huazhong University of Science and Technology, Wuhan, 430022 China

**Keywords:** Piperlongumine, Glioma, Tumor microenvironment, ROS generation/elimination, Immunotherapy, PD-1

## Abstract

**Background:**

The failure of novel therapies effective in preclinical animal models largely reflects the fact that current models do not really mimic the pathological/therapeutic features of glioblastoma (GBM), in which the most effective temozolomide chemoradiotherapy (RT/TMZ) regimen can only slightly extend survival. How to improve RT/TMZ efficacy remains a major challenge in clinic.

**Methods:**

Syngeneic G422^TN^-GBM model mice were subject to RT/TMZ, surgery, piperlongumine (PL), αPD1, glutathione. Metabolomics or transcriptomics data from G422^TN^-GBM and human GBM were used for gene enrichment analysis and estimation of ROS generation/scavenging balance, oxidative stress damage, inflammation and immune cell infiltration. Overall survival, bioluminescent imaging, immunohistochemistry, and immunofluorescence staining were used to examine therapeutic efficacy and mechanisms of action.

**Results:**

Here we identified that glutathione metabolism was most significantly altered in metabolomics analysis upon RT/TMZ therapies in a truly refractory and reliable mouse triple-negative GBM (G422^TN^) preclinical model. Consistently, ROS generators/scavengers were highly dysregulated in both G422^TN^-tumor and human GBM. The ROS-inducer PL synergized surgery/TMZ, surgery/RT/TMZ or RT/TMZ to achieve long-term survival (LTS) in G422^TN^-mice, but only one LTS-mouse from RT/TMZ/PL therapy passed the rechallenging phase (immune cure). Furthermore, the immunotherapy of RT/TMZ/PL plus anti-PD-1 antibody (αPD1) doubled LTS (50%) and immune-cured (25%) mice. Glutathione completely abolished PL-synergistic effects. Mechanistically, ROS reduction was associated with RT/TMZ-resistance. PL restored ROS level (mainly via reversing Duox2/Gpx2), activated oxidative stress/inflammation/immune responses signature genes, reduced cancer cell proliferation/invasion, increased apoptosis and CD3^+^/CD4^+^/CD8^+^ T-lymphocytes in G422^TN^-tumor on the basis of RT/TMZ regimen.

**Conclusion:**

Our findings demonstrate that PL reverses RT/TMZ-reduced ROS and synergistically resets tumor microenvironment to cure GBM. RT/TMZ/PL or RT/TMZ/PL/αPD1 exacts effective immune cure in refractory GBM, deserving a priority for clinical trials.

**Supplementary Information:**

The online version contains supplementary material available at 10.1186/s13046-023-02686-1.

## Introduction

Glioblastoma multiforme (GBM) is the most aggressive and malignant primary brain tumor with a mean survival of 12 months [1] and six molecular subgroups characterized by distinct mutations and DNA methylation profiles, belonging to WHO grade IV [2]. The standard care of GBM, surgical resection followed by radiotherapy plus concomitant and maintenance TMZ (RT/TMZ), remains most effective but can only slightly prolong the median survival for 2–3 months [3, 4]. While many novel therapies, such as immunotherapy [5–7] and oncolytic virotherapy [8], have shown great promise in animal studies but not in clinical trials. Complete surgical removal of GBM cells is nearly impossible due to the highly invasive feature of GBM [9, 10], and therefore the tumor rapidly relapses; meanwhile, GBM cells evolve during RT/TMZ therapy and can acquire “multidrug resistance” (MDR) [2, 11], greatly limiting their efficacy. Thus, a major and practical strategy that can extend patient survival is to improve RT/TMZ efficacy with sensitizing drugs, found it was really difficult in clinical trials. One of the major reasons is that current preclinical GBM animal models do not faithfully recapitulate the key pathological characteristics and therapeutics of GBM [2, 12], and therefore the synergized efficacy of most combined regimens in animal models cannot be verified clinically. Clearly, the use of more representative GBM animal model in drug discovery will produce more reliable efficacy data that can be successfully translated to humans.

In order to facilitate mouse-to-human translation, we have recently developed a highly stable preclinical mouse GBM model, named as G422 triple-negative GBM (G422^TN^-GBM) based on its genetic combinations of IDH1/2^WT^ chromosome1/19^Intact^ TERT-promoter^WT^ with ATRX^Mutant^ Trp53^Mutant^ [13]. This model is different from others as G422^TN^ cells have been maintained for in vivo passaging and in high aggressiveness. We have optimized the G422^TN^-GBM mouse model [13, 14], making it faithful to recapitulate the therapeutic responses of human GBM. In this model, the standard surgery/RT/TMZ regimen or RT/TMZ are most effective but only slightly increases mouse survival time with a comparable efficacy of human GMB [13]. With this highly refractory G422^TN^-GBM model, we have tested the synergistic efficacy of RT/TMZ plus several potential sensitizing drugs (including mannose, metformin or disulfiram/copper gluconate) and demonstrated that only mannose was effective, verifying the value of this model in potential drug screening [13].

In this study, we aim to find new candidates for GBM treatment by overcoming RT/TMZ resistance most relevant to reactive oxygen species (ROS) scavengers. Since targeting ROS by either ROS inducers or antioxidant inhibitors to amplify ROS cytotoxicity has become an important therapeutic strategy for many malignant tumors [15, 16], while piperlongumine (PL) kills cancer cells via selectively inducing ROS [17], we tested the synergistic efficacy of RT/TMZ in combination of PL. Indeed, RT/TMZ/PL treatment significantly prolonged overall survival (OS) in the G422^TN^-GBM model and achieved cure by reshaping pro-inflammatory immune microenvironment, in which M1-like tumor-associated macrophages (TAMs) and CD8^+^ T cells were increased depending on ROS accumulation. Furthermore, RT/TMZ/PL substantively enhanced immunotherapy efficacy of anti-PD1 antibody (αPD1).

## Materials and methods

### Animals, murine G422^TN^-GBM cells and orthotopic G422^TN^-mouse model

**Fig. 1:**
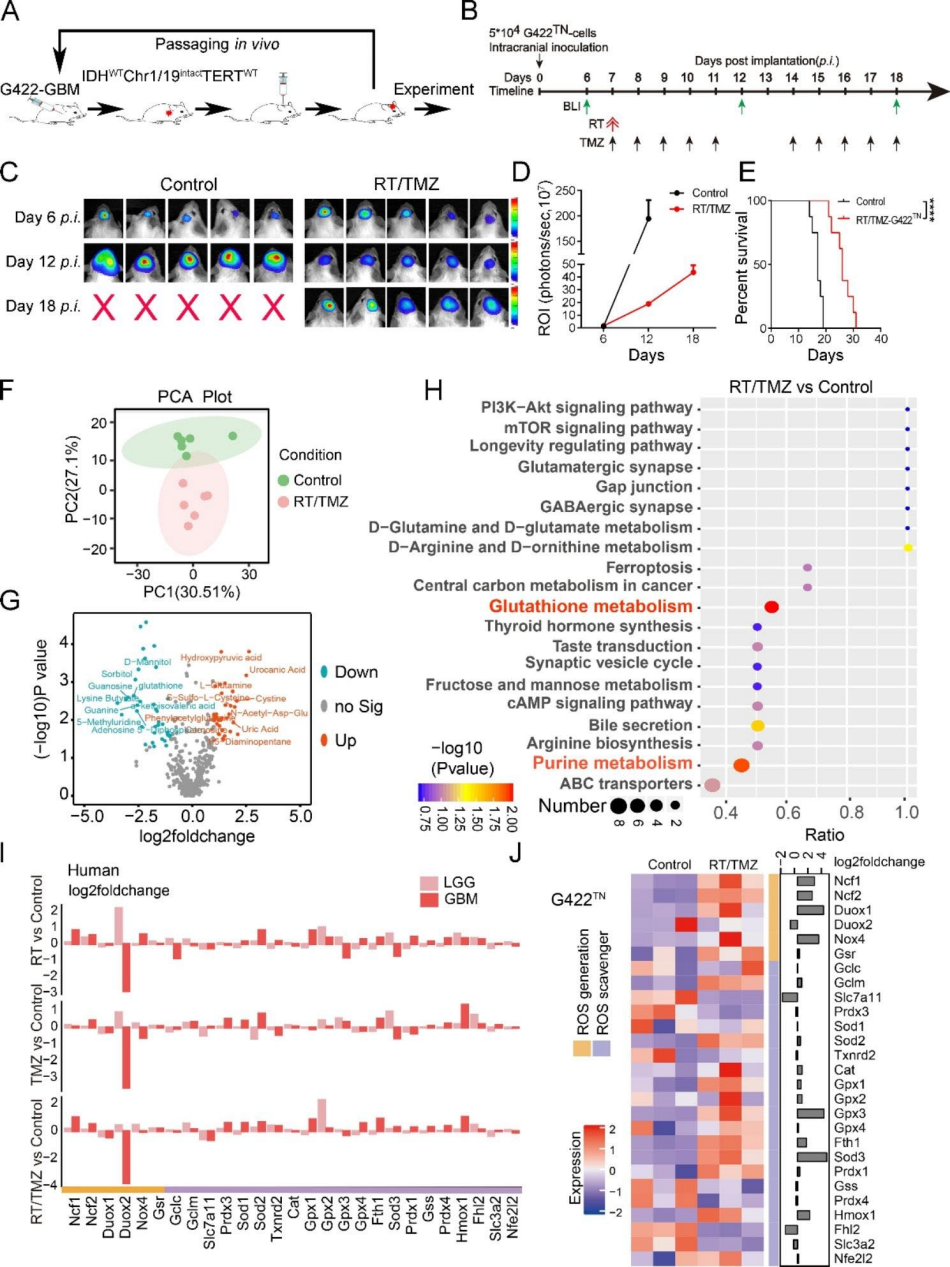
Early alteration of glutathione metabolism restricts RT/TMZ efficacy in orthotopic G422^TN^-tumor. **A** Schematic diagram of orthotopic G422^TN^-GBM mouse model establishment. **B** Schematic diagram depicting the bioluminescent images (BLI) once every six days started on day 6 *p.i.* and RT/TMZ regimen started on day 7 *p.i.* RT, a single dose of 10 Gy-whole brain irradiation (WBI); TMZ, 10 doses of TMZ during one therapeutic course with one oral gavage of 50 µg TMZ per gram of body weight in each dose. **C** and **D** Representative bioluminescent images and statistical analysis of the ROI values of the intracranial tumors in control and RT/TMZ-treated group monitored before and after RT/TMZ treatment (on day 6, 12 and 18, n = 8/group). **E** The Kaplan-Meier survivals of the G422^TN^-mice with RT/TMZ treatment and control group (n = 8/group). **F** The PCA plot of metabolomic data in control and RT/TMZ treatment. **G** The volcano plot of RT/TMZ treatment vs. control metabolites. **H** The top 20 differential pathways between the Control and RT/TMZ identified by the KEGG pathway enrichment analysis. **I** Gene expression profile of ROS generating and scavenging genes in CGGA database including RT treatment vs. control, TMZ treatment vs. control, and RT/TMZ treatment vs. control. **J** Gene expression profile of ROS generating and scavenging genes in control and RT/TMZ treatment. (****P < 0.0001)

Adult male Kunming mice (18–22 g) were purchased from the Experimental Animal Centre, Huazhong University of Science and Technology (HUST). The mice were group housed in the Animal Core Facility of Tongji Medical College with a 12-h light/dark cycle with ad libitum access to food and water. All animals handling and experiments were performed in accordance with the NIH guidelines and the ARRIVE guidelines, and approved by the Institutional Ethics Committees of HUST ([2019] IACUC Number: 2907). The G422^TN^-cells are highly invasive cells purified from orthotopic murine G422-tumors and whole-genome sequencing identify its genotypes matching to triple-negative subtype of human GBM [13]. The orthotopic G422^TN^-GBM tumors in syngeneic Kunming mice possess typical pathological features of human GBM (i.e., highly aggressiveness, necrosis and microvessel hyperplasia) and are molecularly characterized by Vimentin^Hi^GFAP^Lo^CD3^−^. G422^TN^-GBM cells can only survive and be passaged *in viv*o, a condition known to better maintain the tumorigenicity and overall phenotype of cancer cells. Following an optimized protocol, i.e., subcutaneous-intracranial model system, we have established a stable orthotopic G422^TN^-GBM murine model suitable for preclinical drug verification [13]. Briefly, mice were anesthetized with an intraperitoneal injection of chloral hydrate (350 mg/kg) and xylazine (10 mg/kg). Freshly isolated 5 × 10^4^ G422^TN^-cells (in 1 µl PBS) from subcutaneous tumor were microinjected by 10-µl Hamilton syringe into right striatum of mouse brain (coordinates: 0.5 mm anterior and 2.0 mm lateral from the bregma, and 3.5 mm deep from the skull surface) or right superficial cerebral cortex (0.5 mm anterior, 2.0 mm lateral and 2.0 mm deep) to prepare non-surgical or surgical preclinical G422^TN^-mouse models correspondingly as previously described (Fig. 1A). No unforeseen adverse events were observed in all experimental animals.

### Temozolomide concurrent chemoradiotherapy and TMZ adjuvant chemotherapy (RT/TMZ regimen)

G422^TN^-mice accepted therapy starting on day 7 post implantation (*p.i.*) as previously described, a suitable window evaluating therapeutic efficacy [13]. For RT therapy, G422^TN^-mice in their prone position were subjected to whole brain irradiation (WBI) with one dosage of 10 Gy (5 Gy irradiation-5 min interval-5 Gy irradiation) with an X-ray irradiator (RS-2000 pro, Rad Source Technologies, USA) under fixed parameters (160 KV, 25 Ma, 1 Gy/48.4 s). During WBI, a reflector was used to improve the uniformity of irradiation and the other parts of the mouse body were blocked from irradiation with a 3 mm lead plate. For TMZ therapy, TMZ (AbMole, China) was dissolved in 0.5% CMC-Na at a concentration of 5 mg/ml, and was administered to mouse (50 mg/kg) daily via oral gavage. The complete TMZ therapy course was: 5 day TMZ-2 day interval-5 day TMZ. On the first day RT/TMZ regimen, TMZ was administrated 2 h after radiation. Animals were observed daily and euthanized when they demonstrated morbidity signs including hunched posture, lethargy, difficulty ambulating, and weight loss.

### Bioluminescent imaging (BLI)

G422^TN^-cells have been previously infected with luciferase-expressing lentivirus and thus can be used for tumor volume monitoring by BLI method in vivo [13]. BLI of intracranial G422^TN^-tumors was performed with an animal in vivo optical imaging system (Spectral LagoX, USA) 10 min after a single intraperitoneal injection of 0.2 ml of sterile D-luciferin (15 mg/ml in PBS, Cayman Chemical Company, USA). The region of interest (ROI) values measured by using Amiview software (Spectral Instruments Imaging Company, USA) were used for statistical analysis of optical density values.

### Metabolomics data analysis

Metabolomics data were collected as previously described [13]. Briefly, adult mice bearing subcutaneous G422^TN^-tumors of 0.8-1 cm in diameter were treated with vehicle (control) or RT/TMZ (total body irradiation, 10 Gy; plus TMZ, 50 mg/kg/d) for 2 days and then the tumors were isolated for metabolomics. More than 200 mg of fresh tumor tissues were used for the measurement of targeted metabolites by LC-MS analysis (Novogene Bioinformatics Technology Co., Ltd). The analysis software SCIEX OS (version1.4) was applied to peak integration. The standards to defined differential metabolites were absolute value of fold change > = 2 and adjusted p value < 0.05. All differential metabolites were shown in Supplementary Table 1.

### Surgery and its combined regimen

G422^TN^-tumors in superficial cerebral cortex of mice were subjected to surgery or surgery combined regimen as previously described [13, 14]. Briefly, intracranial tumor was fully exposed by removing skull cap of 5 mm-in diameter centered with the original injection site. The tumor mass was macroscopically completely removed with micro-forceps under a stereo microscope until apparent white walls in the surgical cavity, indicating the reaching of normal brain parenchyma region. Hemostasis was accomplished by using gel foam. The effectiveness of this protocol has been verified in previous studies [13]. Less than 5% of surgical deaths occurred within 48 h after the surgery. For surgery combined regimen, other therapies were started on day 8 *p.i*. (i.e., one day after surgery). Animals were euthanized when they demonstrated morbidity signs including hunched posture, lethargy, difficulty ambulating, and weight loss.

### Piperlongumine, glutathione, mannose and anti-PD-1 antibody (αPD1) treatment

Piperlongumine (Selleck Chemicals, China) was dissolved in tween-80/water (10/90, v/v) at a final concentration of 0.5 mg/ml, and was administered to mice (5 mg/kg/d) daily via intraperitoneal injection according to corresponding therapeutic schedules. Glutathione (Selleck Chemicals, China) was dissolved in normal saline at a final concentration of 40 mg/ml, and was administered to mice (400 mg/kg/d) daily via intraperitoneal injection corresponding therapeutic schedules. Mannose (AbMole, China) was dissolved in 200 ml sterile water (20% (w/v)), which each mouse received a total amount of 40 g via normal drinking per week [13], supplemented with one dose of 40 mg by oral gavage three times per week. For αPD1 immunotherapy, αPD1 (clone RMP1-14, Bio X Cell) or its isotype control was administered to mice via intraperitoneal injection with a first dose of 400 µg/mouse and other doses of 200 µg/mouse every other day according to corresponding schedules (6 doses in total). Control animals received equivalent doses of isotype murine IgG according to the same dosing schedule.

### Rechallenge assay

G422^TN^-mice survived over 100 days *p.i.* (i.e., long-term survival, LTS) were subjected to rechallenge assay [13]. 5 × 10^4^ G422^TN^-cells (same as the first implantation) were microinjected into left striatum of LTS-mice (0.5 mm anterior and 2 mm lateral from bregma, and 3.5 mm deep from the cortical surface). Age-matched naïve mice were used as controls by implanting same amounts of G422^TN^-cells into left striatum. Without any treatment, intracranial G422^TN^-tumor was measured by BLI on day 7 *p.i.* and the survival time of mice was recorded. Animals were euthanized when they demonstrated morbidity signs including hunched posture, lethargy, difficulty ambulating, and weight loss.

### ROS level measurement

Orthotopic G422^TN^-mice were treated for three days (i.e., from day 7 to day 9 *p.i*.) and sacrificed 6 h post the last drug injection. Whole brains from each group were isolated and immediately frozen in optimal cutting temperature compound, and then cut into 20 μm-thick frozen sections by a cryostat microtome (Leica Biosystems). The ROS level was detected using dihydroethidium (DHE) staining. In brief, frozen brain tissues containing G422^TN^-tumor were incubated with 8 µM of DHE (Sigma, D7008) for 60 min at 37 °C. Cell nuclei were stained with DAPI (1:500, Roche, 216,276) for 5 min. After thrice PBS rinse, the sections were cover-slipped with fluorescent mounting medium (Southern biotech, 0100-01) and photographed using the Olympus IX-73 microscope connected to Olympus DP80 photographic equipment (Olympus, Japan) at 200×magnification under same conditions. The fluorescence of DHE and DAPI was measured at an excitation wavelength of 535 nm and 364 nm.

### Hematoxylin-eosin (H&E) staining and immunohistochemistry (IHC)

Paraffin-embedded brain slices were used for H&E staining and IHC analysis as previously described [18]. Briefly, 4 μm-thick brain slices were deparaffinized, rehydrated, endogenous peroxidase blocked, antigen-retrieved, blocked with 5% BSA, incubated with primary and corresponding secondary antibodies (Polink-1 HRP DAB Detection System, ZSGB-BIO, China), and the colorimetric end products were produced by applying diaminobenzidine tetrachloride. Primary antibodies were anti-Ki67 (1:100, ab16667, Abcam, UK), anti-CD3 (1:2000, ab237721, Abcam, UK), anti-CD4 (1:2000, ab183685, Abcam, UK), anti-CD8 (1:2000, ab209775, Abcam, UK), and anti-FoxP3 (1:100, #12,653, CST, USA). Whole brain images were obtained by scanning the brain sections at 200×magnification with an automatic slice scanning system-SV120 (Olympus, Japan). Statistical analysis used data from 6 slices from 6 mice brains per group. The data were expressed as mean number of positive cells per mm^2^ microscopic field.

### RNA extraction and quantitative real-time PCR (qRT-PCR)

Following the manufacturer’s instructions, total RNA was extracted from subcutaneous tumor tissues using TRIzol reagent (15596-026, Invitrogen, Carlsbad, CA, USA)0.1 µg of extracted RNA was used for cDNA synthesis using HiScriptR III RT SuperMix for qPCR (+ gDNA wiper) (R323-01, Vazyme, China) according to the manufacturer’s instructions. qRT-PCR specific for ROS generation signature genes (*Ncf1, Ncf2, Duox1, Duox2, Nox4, Gsr*), ROS scavenger signature genes (*Gclc, Gclm, Slc7a11, Prdx3, Sod1, Sod2, Txnrd2, Cat, Gpx1, Gpx2, Gpx3, Gpx4, Fth1, Sod3, Prdx1, Gss, Prdx4, Hmox1, Fhl2, Slc3a2, Nef2l2*), antigen presentation signature genes (*B2m, Calr, Canx, Nlrc5, Pdia3, Psmb8, Psmb9, Tap1, Tap2, Tapbp*), IFN gamma signature genes (*Ifngr1, Ifngr2, Jak1, Jak2, Stat1, Stat3, Stat5a, Stat5b, Tyk2*) and inflammatory response signature genes (*Inhbe, Il2rb, Cxcl15, Il1a, Il7r, Ccl20, Il1b, Cxcl3, Il6, Cxcl2*) were performed on Quantagene q225 fluorescent quantitative PCR system. The primers of the above genes were listed in supplementary Table 2. Data were analyzed using 2^−ΔΔCT^ method and normalized to β-actin.

### Terminal deoxynucleotidyl transferase dUTP nick-end labeling (TUNEL) assay

The in situ apoptosis detection kit (Roche, 11,684,817,910) was used, according to the manufacturer’s instructions, to assess the level of apoptosis in paraffin-embedded tissue sections. Six fields with apoptotic cells were observed in each specimen at 200×magnification. The data were expressed as mean number of apoptotic cells per mm^2^ microscopic field.

### Flow cytometric analysis

Brains and spleens of different groups (Control, RT/TMZ, or RT/TMZ/PL) of mice were harvested and homogenized using enzymatic (1.5 mg/mL collagenase IV, 200 U/mL DNase I, HBSS with calcium and magnesium) and mechanical tissue disaggregation. The solution was filtered twice over a 70 μm nylon filter and centrifuged for 5 min at 1200 rpm, 4℃. The pellet was resuspended in 3ml 70% standard isotonic percoll (SIP, GE Healthcare) and gently overlaid with 3ml of 37% SIP followed by a 3ml layer of 30% SIP, forming a three-layered density gradient (centrifuged at 800 g, 4℃, 30 min without acceleration/braking). The 70/37% interphase containing immune cells was collected and centrifuged for 5 min at 1200 rpm, 4℃. The cell pellet was resuspended in pre-cooled PBS and incubated with the antibodies specific to the following proteins at 4 °C for 40 min: CD45-BV510 (BioLegend, 103,137), CD3-FITC (BioLegend, 100,203), CD4-PE-Cy7 (BioLegend, 116,015), CD8-PE (BioLegend, 107,708). Unlabeled cells were used as the blank control. Dead cells were excluded using Zombie Aqua™ Fixable Viability Kit (BioLegend, 423,101). Data acquisition and compensation were performed on ID7000™ full spectrum flow cytometry analyzer (Sony, Japan) and analyzed using FlowJo VX 10.

### RNA sequencing data analysis

Adult mice bearing subcutaneous G422^TN^-tumors (0.8-1 cm in diameter) were respectively treated with vehicle (control), PL (5 mg/kg/d), RT/TMZ (total body irradiation, 10 Gy; plus TMZ 50 mg/kg/d) or RT/TMZ/PL (RT/TMZ puls PL) for 2 days, and then the subcutaneous tumors were isolated for transcriptomics. Tumor tissue were subjected to total Mrna isolation, Cdna libraries construction and sequencing at Illumina HiSeq sequence platform (PE150) with 6G clean data by Novogene Bioinformatics Institute (Shanghai, China). For bulk RNA sequencing data under different treatments including control, PL, RT/TMZ, and RT/TMZ/PL, we firstly removed low quality bases and sequences using TrimGalore (https://www.bioinformatics.babraham.ac.uk/projects/trim_galore/). Then, remained sequences were aligned to mm10 mouse genome reference using STAR [19]. Gene expressions were quantified using RSEM [20] to obtain counts and FRKM of per sample (Supplementary Table 3). We performed differential expression analysis using DESeq2 (version 1.28.1) [21]. The standards to define DEGs were adjusted *P* value < 0.05 and absolute value of fold change > = 2 (Supplementary Table 4).

### TCGA and CGGA cohorts

The cancer genome atlas (TCGA) lower grade glioma (LGG) and GBM gene expression data were obtained from the UCSC Xena. Gene expressions levels of all samples were measured by FPKM. We also downloaded raw counts for differential genes analysis using DESeq2 [21], and clinical information for survival analysis. Bulk RNA sequencing data of LGG and GBM were obtained from the Chinese Glioma Genome Atlas (CGGA) database, which contained gene expression data processed by the CGGA pipelines. In addition, clinical information including treatment options and survival were downloaded for further analysis.

### Gene set and differential metabolites enrichment analysis

All pathway enrichment analyses were based on KEGG database resources [22]. The ClusterProfiler R package [23] was used to enrich pathways and visualize enrichment results. Similarly, differential metabolites were enriched by KEGG analysis and visualized by the ggplot2 R package. In this study, we constructed many signature scores using single-sample GSEA (ssGSEA) method [24]. TAMs signature genes were provided in Supplementary Table 5.

### Correlation analysis and immune cell infiltration estimation

We calculated Pearson correlations of the RT/TMZ/PL signature score with the antigen presentation signature, the IFN gamma signature and the inflammatory response signature, respectively. Immune cell proportion was calculated with MCP-counter function in the MCP R packages [25].

### Statistical analysis

Animal survival was analyzed by the Kaplan-Meier estimate and compared using a log-rank (Mantel Cox) test. Two-tailed unpaired t-test was used to analyze the differences between two unpaired groups. Paired t-test was used to analyze two paired groups. One-way analysis of variance (ANOVA) with post-hoc Dunnett’s test was used to compare one-factor variable experiments among multiple groups. All values were reported as mean ± SEM. Differences were considered significant at a value of *P* < 0.05. Patient’s Kaplan Meier survival curves were used to assess differences in overall survival times between the high oxidative stress signature group and the low oxidative stress signature group. Survival curves were constructed by ‘gsurvplot’ function in the R package survminer (version 0.4.2) from Bioconductor. GBM patients in TCGA and CGGA database were classified into high oxidative stress signature group or low oxidative stress signature group using the survcutpoint function in the R package survminer. Descriptions for statistical analysis were shown in each corresponding figure legend. Statistical analysis was performed using R software (version 4.0.2) and GraphPad Prism software.

## Results

### Limited efficacy of RT/TMZ to orthotopic G422^TN^-tumor is associated with early alteration of glutathione metabolism

In order to obtain reliable preclinical data for drug evaluation, we have established a highly refractory orthotopic G422^TN^-GBM mouse model. Following previously optimized protocol (Fig. 1A), inoculating 5 × 10^4^ G422^TN^-cells into striatum caused rapid tumor growth as visualized on day 6, 12 and 18 post implantation (*p.i.*) by in vivo BLI with or without therapy (Fig. 1B and D). The tumors maintained rapid growth during RT/TMZ therapies, possessing a faster growth during 12th -18th day than 6th -12th reflected by the ROI slope (Fig. 1D). Meanwhile, all G422^TN^-mice died within 15 days after discontinuing treatment (Fig. 1E). These results verified the malignancy and RT/TMZ-resistance of G422^TN^-tumor, thus the curative effect of RT/TMZ gradually deteriorating.

Then, we aim to find the major mechanism that confers G422^TN^-tumor resistance to RT/TMZ by utilizing metabolomics data. Comparing the global metabolites of subcutaneous G422^TN^-tumors, the results showed distinct differences between RT/TMZ and control group (Fig. 1F and Supplementary Fig. 1), indicating the metabolic status changes. In total, 71 differential metabolites (DMs) were detected by bioinformatics analysis (fold change > 2 and *P* value < 0.05) (Fig. 1G), and further KEGG enrichment analysis based on these DMs revealed Glutathione metabolism to be the most significantly altered metabolic pathway (Fig. 1H). Since modulating ROS represents a strategy to kill MDR cancer cells [11], while GSH is well known for inhibiting therapeutic efficacy by neutralizing ROS in various cancers including GBM [26], we speculated that the early and prominent alteration of GSH metabolism played a major role in limiting RT/TMZ efficacy in G422^TN^-tumors and human GBM.

Furthermore, we analyzed the gene profiles of ROS generation (ROS_gen_) and ROS scavenger (ROS_sca_) signature genes in human glioma by utilizing the Chinese Glioma Genome Atlas (CGGA) database, and stratified the cohort into GBM and lower grade glioma (LGG), with or without therapy (control, RT, TMZ or RT/TMZ). The results revealed that most of ROS_gen_ and ROS_sca_ were upregulated in all therapy groups as compared to their corresponding control groups although differential expression existed among groups (Fig. 1I). Among all therapy groups, RT/TMZ-GBM had most upregulated ROS_sca_ (18/21, including 3/4 *Gpx*) while its ROS_gen_/ROS_sca_ fold changes ratio was the lowest, which were closest to that of RT/TMZ-treated G422^TN^-tumors (Fig. 1J). These results together demonstrated that RT/TMZ reset ROS metabolism in a similar pattern, mainly by upregulating ROS_sca_ including Gpxs, in both G422^TN^-tumors and human GBM.

### PL prominently improves RT/TMZ efficacy to achieve long-term survival and immune cure in G422^TN^-mice

**Fig. 2:**
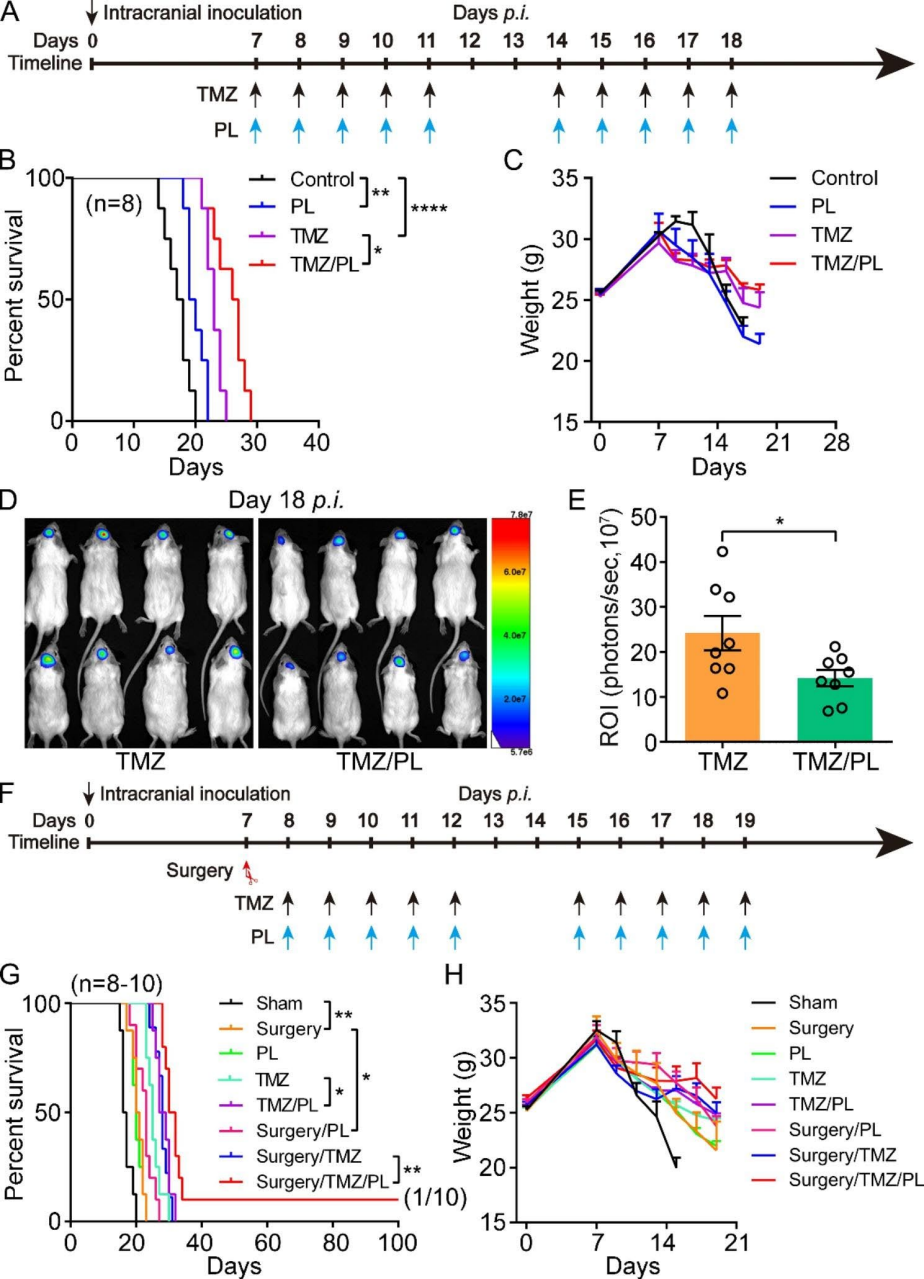
PL prominently improves TMZ and surgery/TMZ efficacy in G422^TN^-mice. **A** Schematic diagram depicting the Piperlongumine (PL), TMZ chemotherapy (TMZ), or their combined regimen started on day 7 *p.i.* PL, 10 doses of PL with once oral gavage of 5 µg/g. **B** and **C** The Kaplan-Meier survivals and body weight changes of the G422^TN^-mice with PL, TMZ, or TMZ/PL treatment started on day 7 *p.i.* (n = 8/group). **D** and **E** Representative bioluminescent images and statistical analysis of the ROI values of the intracranial tumors monitored on day 18 *p.i.* (n = 8/group). **F** Schematic diagram of the surgery, PL, TMZ chemotherapy (TMZ), or their combined regimen started on day 7–8 *p.i.***G** and **H** The Kaplan-Meier survivals and body weight changes of the G422^TN^-mice with surgery, PL, TMZ, or their combined regimen started on day 7–8 *p.i.* (n = 8–10/group). (*P < 0.05; **P < 0.01; ****P < 0.0001)

PL exerts an antitumor effect via reducing GSH selectively to induce ROS accumulation, so we tested whether targeting GSH metabolism via PL may improve RT/TMZ efficacy [27]. PL monotherapy significantly improved OS of G422^TN^-mice although less effective than TMZ monotherapy (Fig. 2A C). Additionally, PL significantly improved efficacy (enhancing OS and reducing tumor size) of TMZ but all G422^TN^-mice died within day 30 *p.i.* (Fig. 2B and E). Further, we tested the efficacy of PL in combination with surgery, as surgery is commonly applied to remove primary GBM in clinic. Surgical removal of microscopically visible tumor mass on day 7 *p.i.* achieved similar efficacy of PL monotherapy (Fig. 2F H). Surgery followed by PL administration significantly improved surgery efficacy, while TMZ/PL showed similar efficacy of surgery/TMZ (Fig. 2G). Surgery/TMZ/PL further improved OS of G422^TN^-mice as compared to surgery/TMZ, and achieved LTS in 10% of G422^TN^-mice (1/10) while no other PL combined therapies could extend G422^TN^-mice survival to up 35 days (Fig. 2G).

**Fig. 3:**
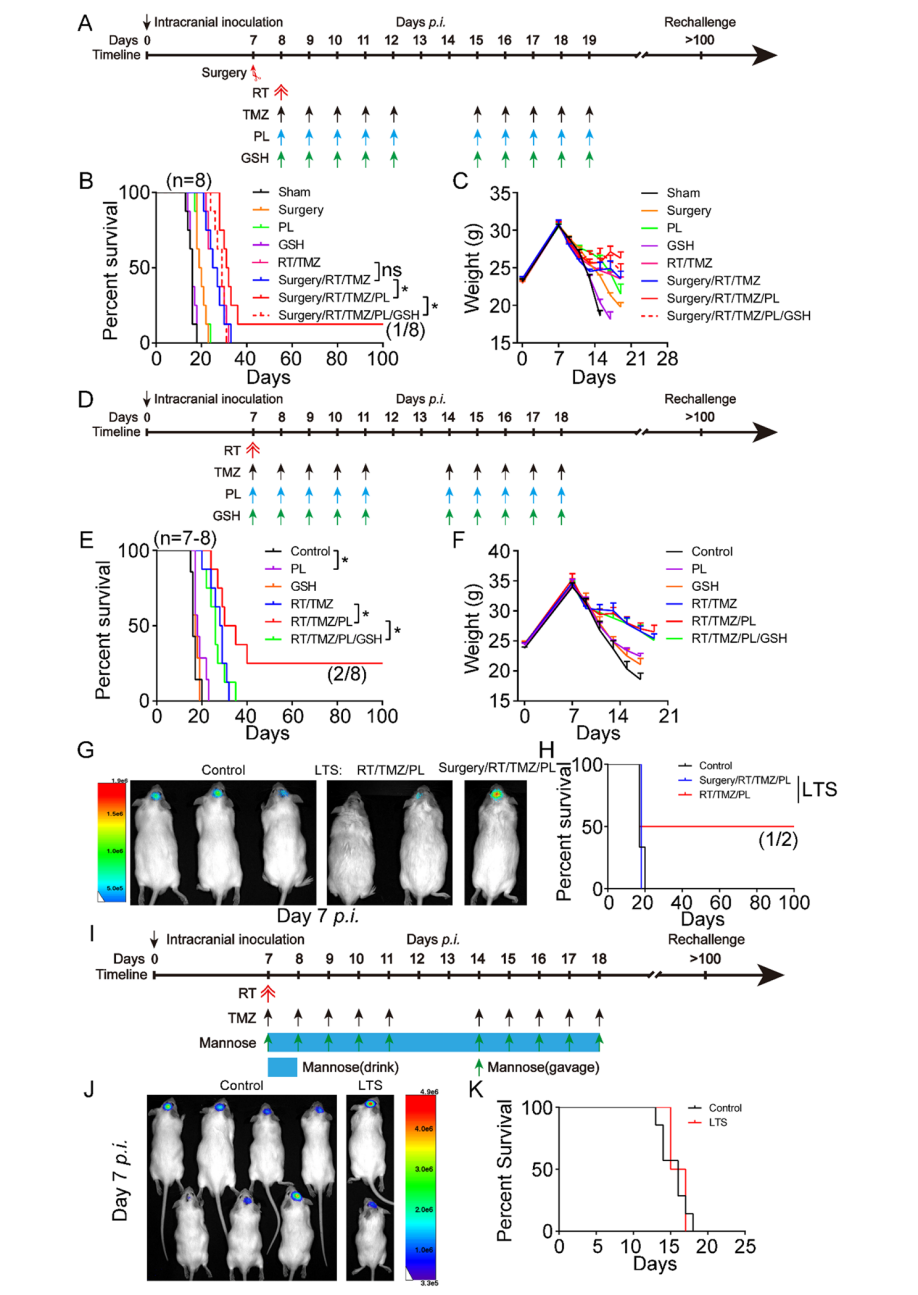
PL prominently improves surgery/RT/TMZ and RT/TMZ efficacy to achieve long-term survival and immune cure in G422^TN^-mice. **A** Schematic diagram depicting the surgery, Piperlongumine (PL), RT, TMZ chemotherapy (TMZ), GSH, or their combined regimen started on day 7–8 *p.i.* GSH, 10 doses of GSH with once oral gavage of 400 µg/g. **B** and **C** The Kaplan-Meier survivals and body weight changes of the G422^TN^-mice with surgery, PL, RT, TMZ, GSH, or their combined regimen started on day 7–8 *p.i.* (n = 8/group). **D** Schematic diagram of the PL, RT, TMZ, GSH, or their combined regimen started on day 7 *p.i.***E** and **F** The Kaplan-Meier survivals and body weight changes of the G422^TN^-mice with PL, RT, TMZ, GSH, or their combined regimen started on day 7 *p.i.* (n = 7–8/group). **G** and **H** Representative bioluminescent images and the Kaplan-Meier survivals of control (n = 3), RT/TMZ/PL (LTS, n = 2) and surgery/RT/TMZ/PL (LTS, n = 1) group during rechallenge. **I** Schematic diagram depicting the mannose, RT, TMZ, or their combined regimen started on day 7 *p.i.*. **J** Two of eight G422^TN^-mice achieved LTS in RT/TMZ/Mannose group, and were further subjected to rechallenging assay. BLI showed the presence of G422^TN^-tumor in control (n = 7) and LTS (n = 2) mice. **K** The Kaplan-Meier survival curves showed no difference between control and LTS group during rechallenge phase. (*P < 0.05; ns, not statistically significant)

When PL combined with standard therapy for GBM, the OS of G422^TN^-mice was further improved as compared to surgery/RT/TMZ, with the LTS ascending to 12.5% (Fig. 3A C). Administration of GSH completely abolished surgery/RT/TMZ/PL synergized efficacy (Fig. 3B). Unexpectedly, surgery did not increase the efficacy of RT/TMZ (Fig. 3B), so PL combined with RT/TMZ was considered as the further experimental protocol. Interestingly, RT/TMZ/PL regimen achieved LTS in 25% of G422^TN^-mice (2/8) (Fig. 3D F). GSH completely abolished RT/TMZ/PL synergized efficacy and no G422^TN^-mice survived over 35 days (Fig. 3E), similar to surgery/RT/TMZ/PL regimen (Fig. 3B). The tumor size on day 14 also verified the synergized efficacy of RT/TMZ/PL and the inhibitory effect of GSH (Supplementary Fig. 2). These results demonstrated that PL effectively enhanced RT/TMZ efficacy of G422^TN^-GBM by targeting ROS or GSH.

Finally, all three LTS mice obtained from the above were subject to rechallenge test. 5 × 10^4^ G422^TN^-cells were implanted into left striatum of LTS mice or normal mice (control). BLI at day 7 *p.i.* verified the growth of G422^TN^-tumors in all mice (Fig. 3G). Only one LTS mouse from the RT/TMZ/PL group (1/2) survived over 100 days *p.i.*, while all other G422^TN^-mice died within 20 days *p.i.* (Fig. 3H); when mannose was administered in combination with RT/TMZ (Fig. 3I), there were two LTS mice, both of which died within 20 days when rechallenged (Fig. 3J K), indicating that RT/TMZ/mannose is less effective than RT/TMZ/PL. These results demonstrated RT/TMZ/PL achieved the achievement of immune cure in these LTS mice.

### PL restores RT/TMZ-reduced ROS and augments oxidative damage in G422^TN^-tumor via reprograming ROS modulator genes

**Fig. 4:**
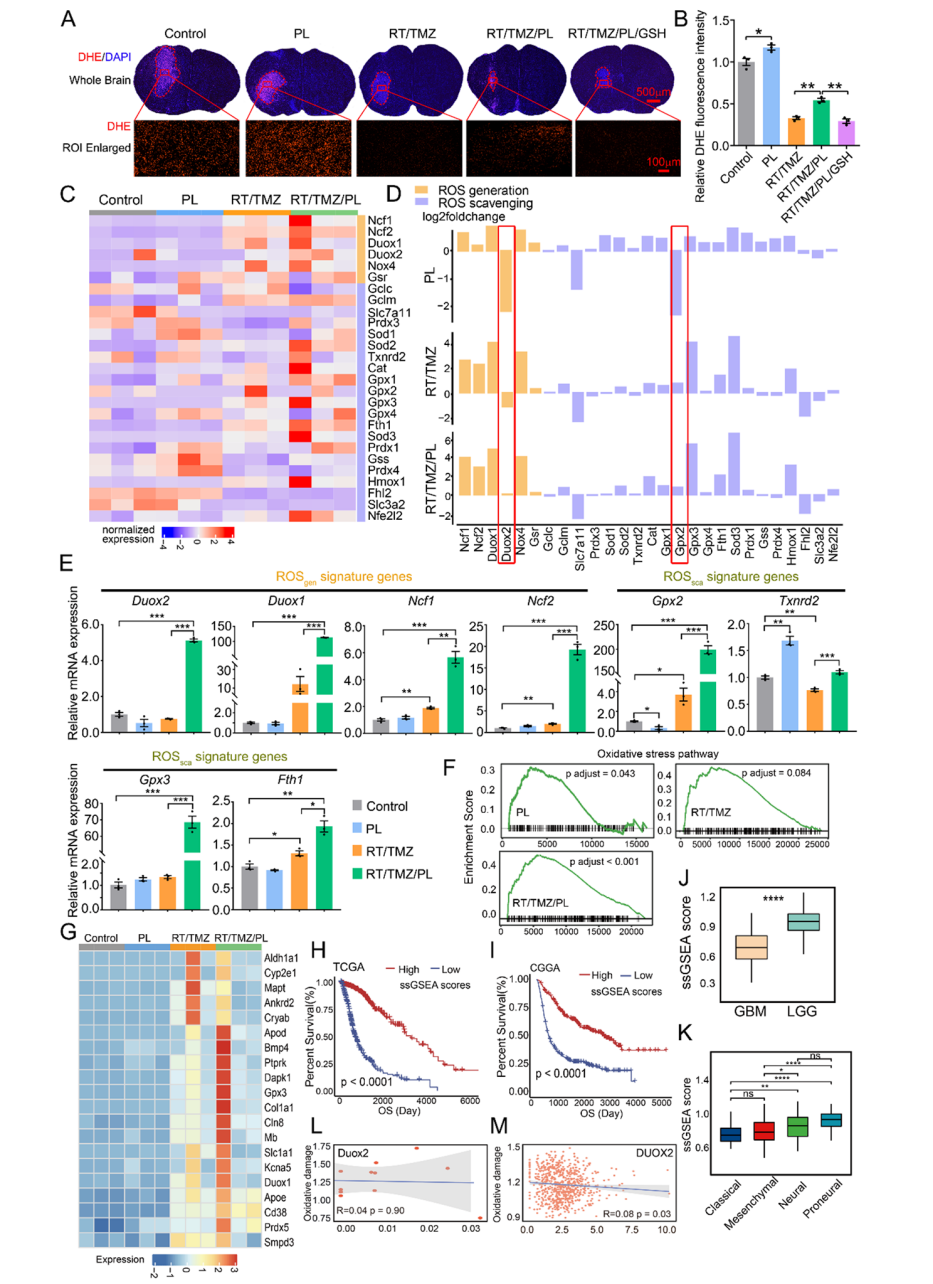
PL restores RT/TMZ-reduced ROS and augments oxidative damage in G422^TN^-tumor via reprograming ROS modulator genes. **A** Representative DHE staining of ROS levels in control, PL, RT/TMZ, RT/TMZ/PL, RT/TMZ/PL/GSH group of G422^TN^-mice. Upper panel: whole brain figures. Scale bar, 500 μm. Lower panel: partial graphs. Scale bar, 100 μm. (n = 3). **B** Statistical analysis of ROS levels in control, PL, RT/TMZ, RT/TMZ/PL, RT/TMZ/PL/GSH group of G422^TN^-mice. (n = 3). **C** Gene expression of ROS generating and scavenging genes of all samples, the bar above the heatmap represents the group information, and the bar on the right of the heatmap represents the category of genes. **D** Fold changes of gene expression of ROS generating and scavenging genes in different comparison (PL treatment vs. control, top) and (RT/TMZ/PL treatment vs. control, bottom). **E** qRT-PCR showing the mRNA expression of ROS_gen_ (*Duox2, Duox1, Ncf1, Ncf2*) and ROS_sca_ signature genes (*Gpx2, Txnrd2, Gpx3, Fth1*). (n = 3). **F** Enrichment plot of ranked genes illustrating the enrichment of oxidative stress in different condition including PL treatment vs. control, RT/TMZ treatment vs. control, and RT/TMZ/PL treatment vs. control. **G** The heatmap of genes related to oxidative stress. **H** and **I** Patient survival in high oxidative signature group vs. low oxidative signature group of TCGA (**H**) LGG&GBM dataset, and CGGA (**I**) LGG&GBM dataset. **J** The boxplot illustrating ssGSEA score of the oxidative signature induced by RT/TMZ/PL treatment in different glioma types of TCGA dataset (Student’s t-tests). **K** The boxplot illustrating ssGSEA score of the oxidative signature induced by RT/TMZ/PL treatment in different GBM subtypes of TCGA dataset (Student’s t-tests). **L** and **M** Correlation between Duox2 expression and the oxidative damage signature in bulk RNA sequencing data of animal models (**L**), and of CGGA database (**M**). (*P < 0.05; **P < 0.01; ***P < 0.001; ****P < 0.0001; ns, not statistically significant)

Since PL-induced ROS accumulation is the major mechanism for its cytotoxicity [17], we first measured ROS level in orthotopic G422^TN^-tumors. DHE staining revealed that ROS level in G422^TN^-tumor tissue was much higher than that of normal brain tissue (Fig. 4A and B), reflecting the rapid growth and high energy metabolism of G422^TN^-cells. PL monotherapy further increased ROS levels, while RT/TMZ therapy prominently reduced ROS level. RT/TMZ/PL restored ROS level compared to RT/TMZ group, while this effect could be completely abolished by supplementing GSH (Fig. 4A, B, Supplementary Fig. 3 and Supplementary Fig. 4).

To explore the mechanisms that balance ROS level, we systemically analyzed ROS metabolism genes by utilizing RNA-seq data of subcutaneous G422^TN^-tumors upon PL, RT/TMZ or RT/TMZ/PL treatments and identified the DEGs between the treatments and control (i.e. PL versus Control, RT/TMZ versus Control or RT/TMZ/PL versus Control) (Fig. 1J, and Supplementary Fig. 5A-B). Among DEGs, we compared the expression profile of the 6 ROS_gen_ and the 21 ROS_sca_ signature genes (Figs. 1J and 4 C, 4D and Supplementary Fig. 5C). PL group showed an apparently different pattern to that of RT/TMZ group, especially the expression of ROS_sca_ genes such as *Gpx2*, *Gpx3*, and *Sod3*. Among all signature genes, only *Gpx2* was reversely downregulated upon PL treatment (indicated by a red box) (Fig. 4D). In RT/TMZ/PL group, only Duox2 was reversely upregulated as compared to RT/TMZ group. While compared to PL group, *Gpx2* and *Duox2* were simultaneously reversed in RT/TMZ/PL group (Fig. 4D), partly explaining that the ROS level of RT/TMZ/PL group was between PL and RT/TMZ group. Quantitative real-time PCR and Western blotting analysis were further used to verify the DGEs of bioinformatics analysis. More than 90% of the DGEs were consistent with the trend of bioinformatics analysis of our RNA-seq data (Fig. 4E and Supplementary Fig. 6A-B). Furthermore, we analyzed the correlation between ROS signature genes and OS of GBM patients by utilizing the CGGA database (Supplementary Fig. 7 and Supplementary Table 6). The results revealed the changes of ROS signature genes (i.e., *Gpx2*, *Duox2*) were in line with our RT/TMZ-treated G422^TN^-tumors.

We then evaluated oxidative stress damaging effect of potential excessive ROS in PL, RT/TMZ or RT/TMZ/PL-treated G422^TN^-tumors. The enrichment score containing all signature genes in oxidative stress pathway showed that PL and RT/TMZ/PL therapy significant positively correlated to oxidative stress. The more significant enrichment score in RT/TMZ/PL group demonstrated that PL and RT/TMZ synergistically escalated oxidative stress (Fig. 4F). To quantitatively evaluate therapeutic effects of RT/TMZ/PL-activated oxidative stress, an enrichment ssGSEA score [24] was calculated based on 20 oxidative stress signature genes (Fig. 4G), which were generated by taking intersection between genes positively regulating the oxidative stress pathways and DEGs upregulated in RT/TMZ/PL group (vs. control). Thus, this normalized ssGSEA score was an oxidative stress signature of RT/TMZ/PL therapy. In clinical cohorts from TCGA and CGGA database, a higher ssGSEA score was associated with better OS (GBM + LGG) (Fig. 4H and I), LGG (vs. GBM) (Fig. 4J) and less malignant GBM subtypes (neural and proneural) (Fig. 4K), supporting that RT/TMZ/PL-activated oxidative stress is a therapeutic parameter. Although ROS generation gene Duox2 was specifically upregualted by RT/TMZ/PL, Duox2 alone did not contributed to oxidative damage (evaluated by ssGSEA score) (Fig. 4L and M), suggesting the increased oxidative damage in RT/TMZ/PL-treated G422^TN^-tumors are likely an imbalance net effect of all ROS generation/scavenging genes.

### PL synergizes RT/TMZ to upregulate inflammatory responses and antigen presentation signatures in G422^TN^-tumors

**Fig. 5:**
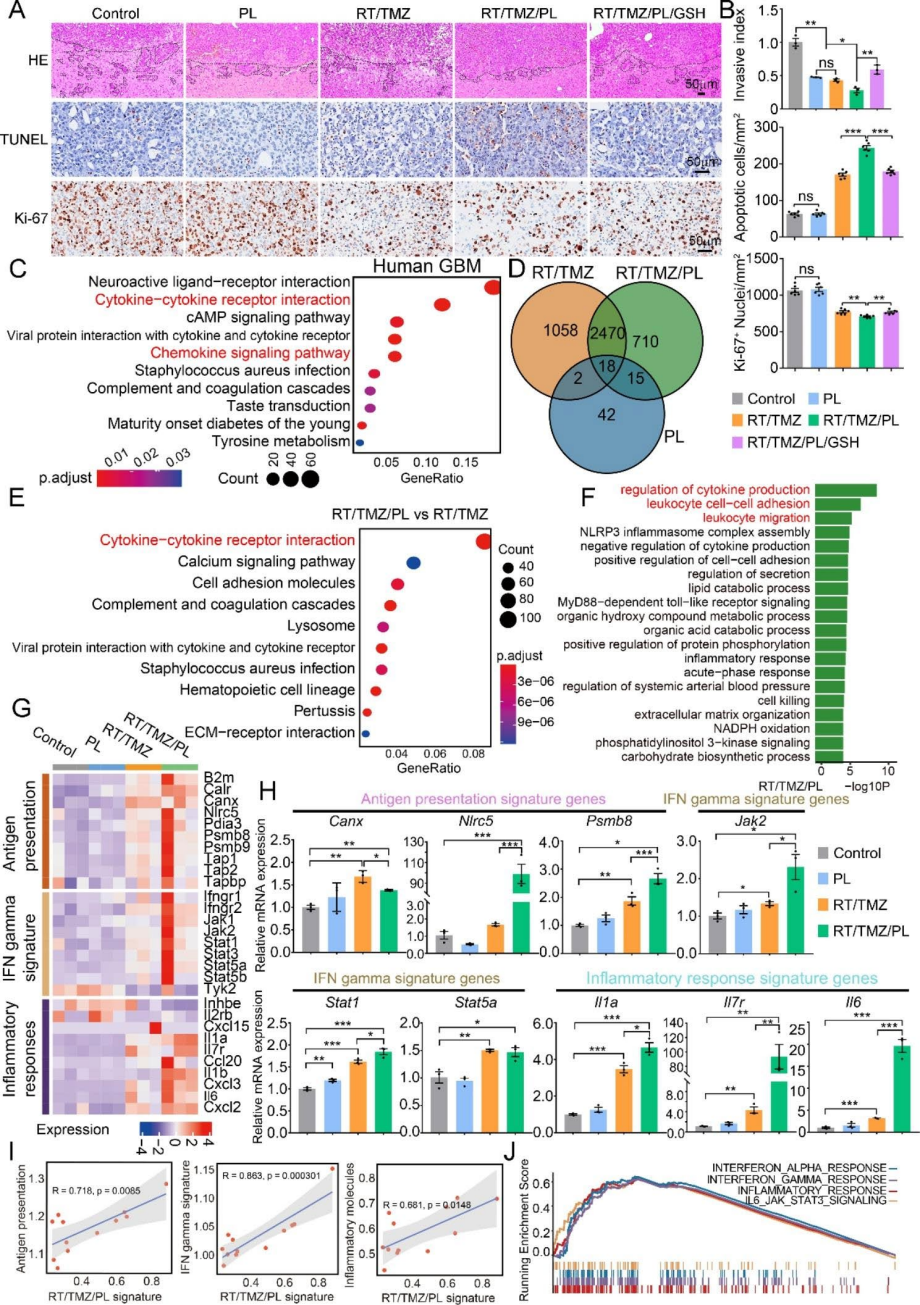
PL synergizes RT/TMZ to upregulate inflammatory responses and antigen presentation signatures in G422^TN^-tumors. **A** and **B** H&E (invasive index, n = 3), TUNEL, KI-67 staining and statistical analysis of G422^TN^-tumor in control, PL, RT/TMZ, RT/TMZ/PL, RT/TMZ/PL/GSH group on day 9 *p.i.* Scale bar, 50 μm. (n = 6) **C** KEGG analysis of upregulated genes in high oxidative signature group in TCGA database. **D** Venn diagram illustrating the differential and overlapping genes upregulated in each group. **E** KEGG analysis of upregulated genes only present in RT/TMZ/PL treatment vs. control. **F** KEGG analysis of upregulated genes present in RT/TMZ/PL treatment vs. RT/TMZ treatment. **G** The heatmap illustrating expression of genes involved in the antigen presentation signature, the IFN gamma signature, and the inflammatory responses signature in different treatments. **H** qRT-PCR showing the mRNA expression of the antigen presentation signature genes (*Canx, Nlrc5, Psmb8*), the IFN gamma signature genes (*Jak2, Stat1, Stat5a*), and the inflammatory response signature genes (*Il1a, Il7r, Il6*). (n = 3). **I** Correlation between the RT/TMZ/PL signature and the antigen presentation signature, the IFN gamma signature, and the inflammatory responses signature, respectively. **J** Enrichment of hallmark pathways for upregulated genes in the RT/TMZ/PL group compared with control. (*P < 0.05; **P < 0.01; ***P < 0.001; ns, not statistically significant)

Then, we verified the effects of RT/TMZ/PL-activated oxidative stress damage by invasive index, TUNEL and Ki-67 staining in intracranial G422^TN^-tumors (Fig. 5A). In control group, Ki-67^+^ cancer cells were prominent, consistent to the rapid growth and high level of ROS in G422^TN^-tumors. PL monotherapy significantly decreased the invasion of cancer cells but did not affect their proliferation and apoptosis (Fig. 5B). RT/TMZ therapy evidently reduced Ki-67^+^ cells, invasive cells and slightly increased TUNEL^+^ cells. PL synergized RT/TMZ to further reduce G422^TN^-cell proliferation and invasion, and increase apoptosis, consistent to the ROS restoration and activated-oxidative stress signature; administration of GSH completely reversed RT/TMZ/PL-synergized effects, verifying the mechanisms of ROS and related oxidative stress damage (Fig. 5B).

A breakthrough efficacy of RT/TMZ/PL in G422^TN^-tumors is immune cure but GSH could completely abolish this cure (Fig. 3E), strongly indicating a causative role of ROS/oxidative stress in immune responses. To explore the underlying mechanisms, we analyzed the potential effects of RT/TMZ/PL-induced oxidative stress signature (i.e., aforementioned ssGSEA score) on human GBM gene expression profiles. KEGG analysis of upregulated genes in high oxidative stress signature GBM group in TCGA database showed that the immune-related pathways, such as cytokine-cytokine receptor interaction and chemokine signaling pathway, were significantly enriched (Fig. 5C). Thus, RT/TMZ/PL-activated oxidative stress signature genes is positively associated with cytokine-induced immune responses in GBM. Then, we exploited RNA-seq data of G422^TN^-tumors to identify RT/TMZ/PL-induced key immune responsive molecules or signals. Upregulated (Fig. 5D) or downregulated (Supplementary Fig. 8A) genes (DEGs) in PL, RT/TMZ or RT/TMZ/PL-treated G422^TN^-tumors (vs. control) were subjected to the KEGG enrichment analysis. The downregulated DEGs in each treatment group were all mainly enriched to neuronal development (Supplementary Fig. 8B); the upregulated DEGs in the three treatment groups, however, were enriched to different pathways. Only in RT/TMZ/PL group, upregulated DEGs were strikingly enriched to immunoregulatory responses such as regulation of cytokine production and leukocyte cell-cell adhesion or migration (Fig. 5E); while in RT/TMZ group or PL group, upregulated DEGs were enriched to immune-unrelated items or metabolic process (Supplementary Fig. 8C and D). Comparing to RT/TMZ group, upregulated DEGs in RT/TMZ/PL were also significantly enriched to cytokine-cytokine receptor interaction (Fig. 5F).

Specifically, many RT/TMZ/PL-upregulated genes were listed in antigen presentation, IFN gamma signature and inflammatory responses (Fig. 5G H and Supplementary Fig. 9A-C). We constructed a RT/TMZ/PL signature using ssGSEA method based on the top 50 upregulated DEGs in RT/TMZ/PL group (Supplementary Table 7). The RT/TMZ/PL signature was positively correlated with the antigen presentation, IFN gamma and inflammatory responses signature, respectively (Fig. 5I). Further, the enrichment score containing all signature genes showed that RT/TMZ/PL therapy significantly positive correlation to IL-6 signaling, interferon and inflammatory responses (vs. control) (Fig. 5J). These results together supported that RT/TMZ and PL synergistically induced G422^TN^-tumor gene reprogramming toward inflammatory immune responses.

### RT/TMZ/PL reshapes immunosuppressive microenvironment to induce CD8^+^ T cells infiltration in G422^TN^-tumors

**Fig. 6:**
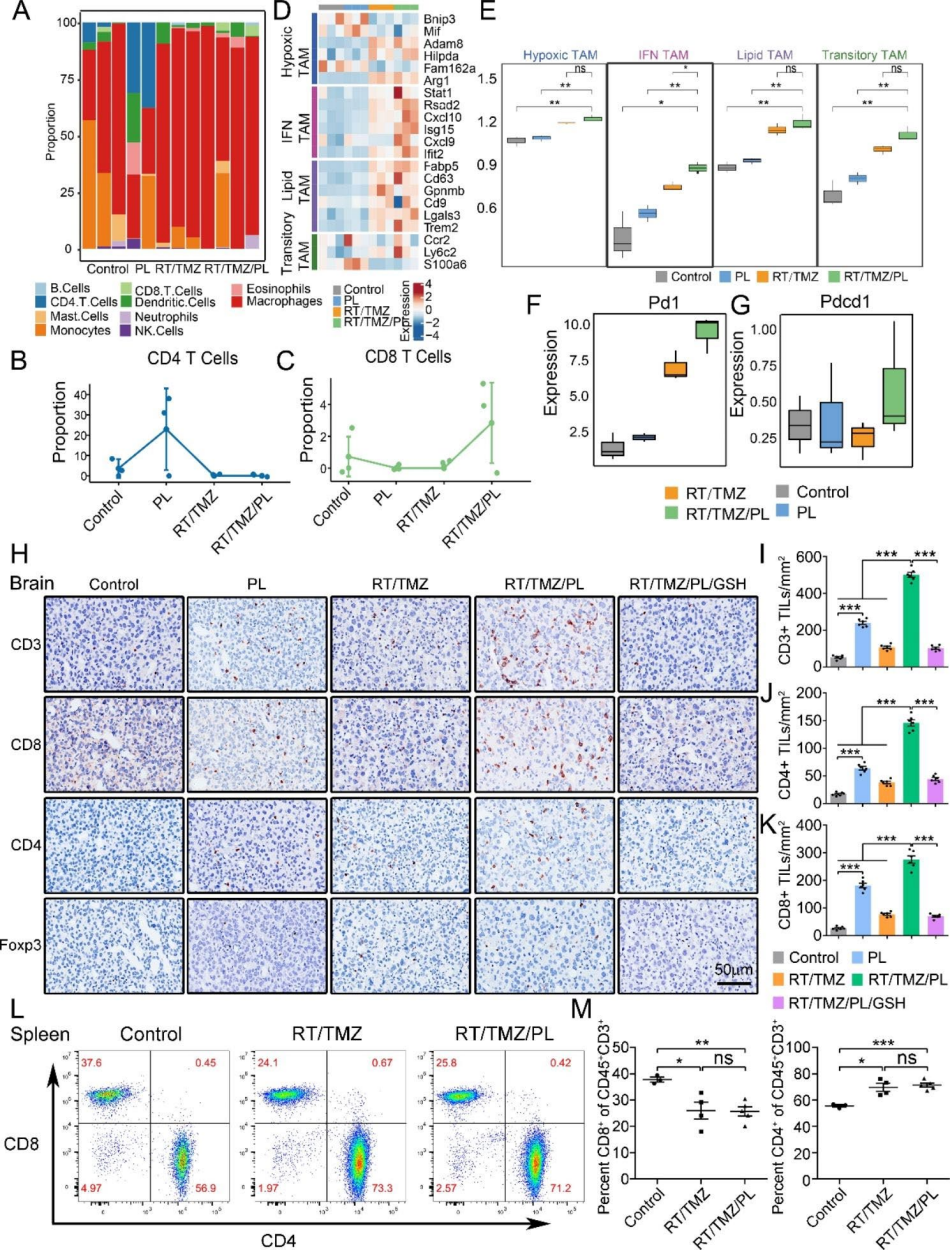
RT/TMZ/PL reshapes immunosuppressive microenvironment to induce CD3^+^/CD4^+^/CD8^+^ T cells infiltration in G422^TN^-tumors. **A** Immune cell proportion analysis in each sample. **B** and **C** The line chart illustrating CD 8 T cell and CD 4 T cell proportion in different treatments. **D** The heatmap illustrating expression of marker genes involved in Hypoxic TAMs, IFN TAMs, lipid TAMs and transitory TAMs in different treatments. **E** ssGSEA score of TAMs subtypes in different treatments. **F** and **G** Expressions of PD1 and PD-L1 in different treatments. **H, I, J** and **K** IHC staining (CD3, CD4, CD8 and Foxp3) and statistical analysis of G422^TN^-tumor in control, PL, RT/TMZ, RT/TMZ/PL, RT/TMZ/PL/GSH group on day 9 *p.i.* Scale bar, 50 μm. (n = 6). **L** and **M** Flow cytometric analysis of T cell gating and statistical analysis in spleen of mice of different groups (control, RT/TMZ and RT/TMZ/PL, n = 3–5). (*P < 0.05; **P < 0.01; ***P < 0.001; ns, not statistically significant)

Successful immune therapy requires a hot tumor microenvironment and CD8^+^ T cells immunity, however, GBM is highly immunosuppressive and deficient in CD8^+^ T cells [28, 29]. We evaluated the effects of PL, RT/TMZ and RT/TMZ/PL on immune cell components/composition in G422^TN^-tumors by analyzing corresponding RNA-seq data: macrophages were predominant in all groups; PL monotherapy mainly increased CD4^+^ T cells and dendritic cells; RT/TMZ mainly increased macrophages; while RT/TMZ/PL mainly increased CD8^+^ T cells (Fig. 6A C and Supplementary Fig. 10). Since tumor-associated macrophages (TAMs) were the majority of immune cells in G422^TN^-tumors and highly heterogeneous, we further analyzed the composition of four TAMs subsets, i.e., hypoxic, IFN, lipid and transitory TAMs, which have been defined by single cell RNA sequencing in mouse glioma models [30]. The heatmap showed that upregulation of IFN TAMs signature genes in RT/TMZ/PL group was more evident compared to other TAMs subsets (Fig. 6D). Among the four subsets, only IFN TAMs were significantly increased in RT/TMZ/PL group compared to RT/TMZ group (Fig. 6E). In addition, the immune inhibitory checkpoint PD-1/PD-L1 pair was upregulated in RT/TMZ/PL group (Fig. 6F and G).

Finally, we verified the effects of RT/TMZ/PL on CD8^+^ T cells infiltration in intracranial G422^TN^-tumors. IHC results demonstrated that CD3^+^ and CD8^+^ T cells were evidently increased in tumor periphery and inner areas of RT/TMZ/PL group compared to all other groups, while CD4^+^ T cells were slightly increased and Treg cells were not altered (Fig. 6H K and Supplementary Fig. 11). Although the few immune cells in the brain limit the use of flow cytometry (FCM) (Supplementary Fig. 12A), FCM of spleen cells in orthotopic G422^TN^-mice showed that CD4^+^ T cells increased and CD8^+^ cells decreased in RT/TMZ and RT/TMZ/PL groups (vs. control) (Fig. 6L, Supplementary Fig. 12B and C). These results together suggested that PL effectively synergized RT/TMZ to turn the “cold” tumor immunosuppressive microenvironment (TIM) to “hot” antitumor microenvironment via IFN TAM-related inflammation, thus promoted CD8^+^ T cells infiltration in GBM.

### αPD1 synergizes RT/TMZ/PL but not RT/TMZ to achieves substantial immune cure in G422^TN^-mice

**Fig. 7:**
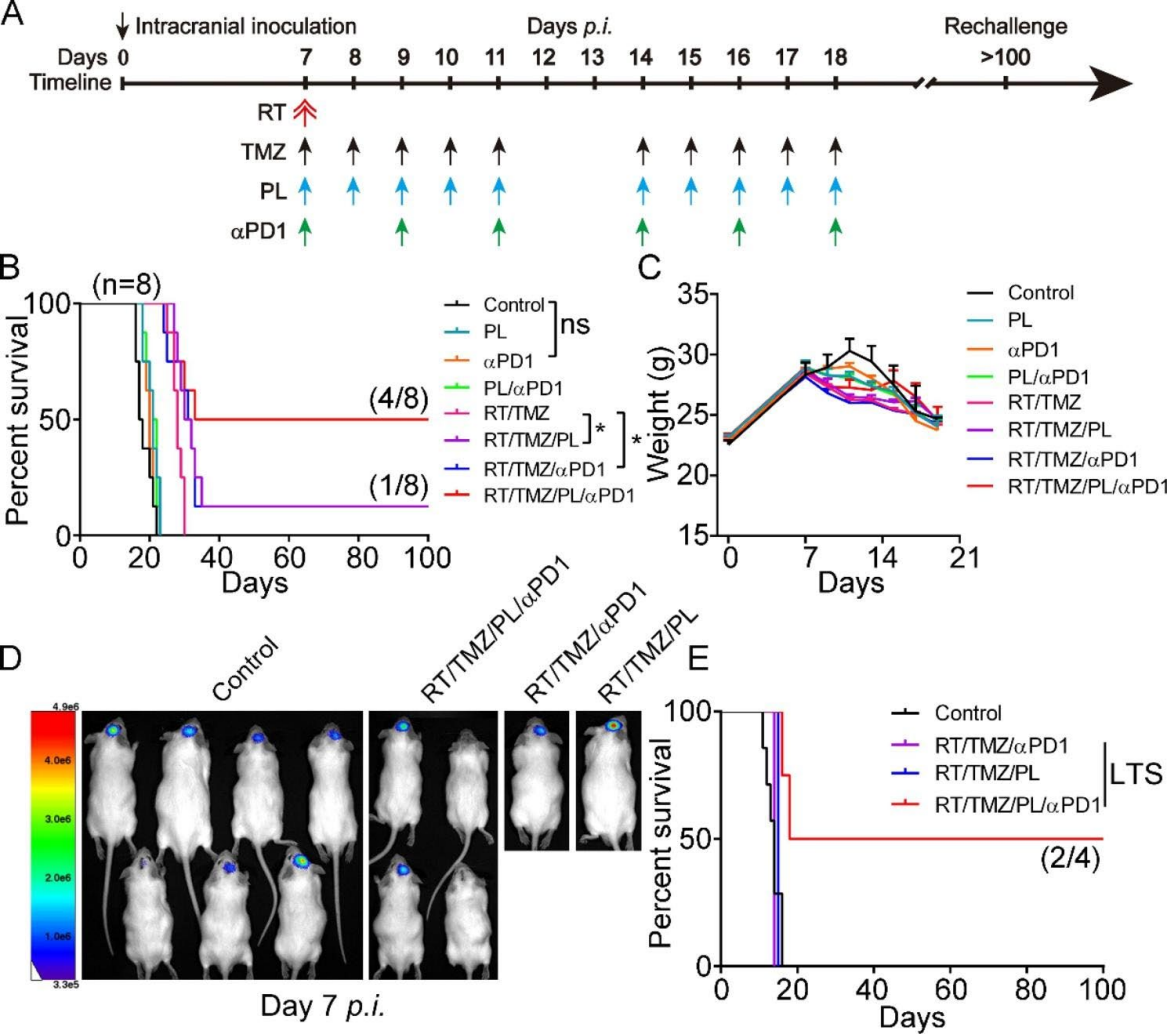
αPD1 synergizes RT/TMZ/PL but not RT/TMZ to achieve substantial immune cure in G422^TN^-mice. **A** Schematic diagram depicting the PL, RT, TMZ, αPD1, or their combined regimen started on day 7 *p.i.* αPD1, 6 doses of αPD1 with once oral gavage of 200 µg/mouse except for first dose (400 µg/mouse). **B** and **C** The Kaplan-Meier survivals and body weight changes of the G422^TN^-mice with PL, TR, TMZ, αPD1, or their combined regimen started on day 7 *p.i.* (n = 8/group). **D** and **E** Representative bioluminescent images and the Kaplan-Meier survivals of control (n = 7), RT/TMZ/PL/αPD1 (LTS, n = 4), RT/TMZ/αPD1 (LTS, n = 1) and RT/TMZ/PL (LTS, n = 1) group during rechallenge. (*P < 0.05; ns, not statistically significant)

Although RT/TMZ/PL combined regimen achieved immune cure in G422^TN^-mice, the efficacy was only around 10% (Fig. 3). The conversion of “cold” to “hot” immune microenvironment by RT/TMZ/PL is a strong favorable indicator for αPD1 immunotherapy [31]. We examined the efficacy of αPD1 in combination with PL, RT/TMZ and RT/TMZ/PL therapies started on day 7 *p.i*. in orthotopic G422^TN^-mice (Fig. 7A C). αPD1 monotherapy did not improve the animal survival (Fig. 7B). αPD1 plus PL showed similar efficacy as PL alone, and had no synergistic effect on OS. αPD1 plus RT/TMZ significantly prolonged OS with one (1/8) animal achieving LTS, similar to the efficacy of RT/TMZ/PL regimen. Surprisingly, αPD1 synergized RT/TMZ/PL achieved 50% of LTS in G422^TN^-mice, significantly prolonging animal survival compared to other groups (Fig. 7B).

All animals achieving LTS from RT/TMZ/PL (one mice), RT/TMZ/αPD1 (one mice) and RT/TMZ/PL/αPD1 (four mice) groups were subjected to further rechallenge assay (Fig. 7D and E). Only two mice (50%) from the RT/TMZ/PL/αPD1 group passed the rechallenge phase (> 100 days). These results demonstrated that RT/TMZ/PL/αPD1 regimen was an effective immunotherapy for highly refractory G422^TN^-GMB.

## Discussion

In the present study, we discovered that prominent glutathione metabolism alteration, in line with early ROS decrease, occurred much earlier than accelerated tumor growth during RT/TMZ therapy by using a novel refractory mouse G422^TN^-GBM preclinical model. Targeting to ROS, PL synergized RT/TMZ, surgery/TMZ or surgery/RT/TMZ to prolong animal survival with LTS, and the former regimen achieving immune cure (survived over 100 days during the rechallenge phase) in orthotopic G422^TN^-mice; otherwise, all G422^TN^-mice died within 35 days. Mechanistically, RT/TMZ/PL synergistically enhanced oxidative stress-inflammation-immunity, increased apoptosis, inhibited cell proliferation, enhanced CD3^+^/CD4^+^/CD8^+^ T-lymphocyte infiltration in G422^TN^-tumors. Importantly, RT/TMZ/PL in combination with αPD1 immunotherapy further doubled the cured G422^TN^-mice (50% LTS and 25% immune cure) compared to RT/TMZ/PL. GSH completely abolished PL-synergized efficacy, demonstrating that PL conquered RT/TMZ-resistance via elevating ROS level. Therefore, RT/TMZ/PL/αPD1 is likely a highly reliable regimen for improving GBM patient OS or even cure.

We discovered that early dysregulated ROS decrease is a key player that restrains RT/TMZ efficacy in GBM. ROS plays dual roles in promoting cancer development and killing cancer cells depending on ROS concentration and cellular context [16, 32]. It is known that cancer cells maintain much higher ROS levels than normal cells, which are critical for its high proliferation rate, accelerated metabolism and tumor progression [16]. Therefore, it is fundamentally important for cancer cells to balance the ROS levels with further oxidative damage that cells can bear [11, 16]. However, how cancer cells deal with such oxidative pressure is complex and remains incompletely understood. Tumor cells are adapted to ROS-driven proliferation under conditions where this oxidative burden pushes redox balance away from a reduced state, achieved by increasing their antioxidant status [11, 16]. It is hypothesized that cancer cells contain a higher balanced ROS/antioxidants level, which contributes to their acquiring of MDR during therapy; on the other hand, further increase ROS by drugs is considered as an important therapeutic strategy to overcome MDR of cancer cells [11]. In our intracranial G422^TN^-GBM model, most G422^TN^-cells on day 9 *p.i.* possessed a much higher ROS level than their neighboring normal cells but cell death was scarce (Figs. 4 and 5), suggesting that G422^TN^-cells had achieved a higher balanced level of ROS/antioxidants along rapid tumor growth (large amounts of Ki-67-positive G422^TN^-cells). PL monotherapy did not significantly increase apoptosis in G422^TN^-cells as expected (Figs. 4 and 5), suggesting that ROS-damaging defense mechanisms were also further escalated. Further, PL only increased the invasion of G422^TN^-cells, might related to its low therapeutic dose [33]. Previous studies have also reported that radiation and chemotherapy including TMZ killed cancer cells in vitro by augmenting ROS/oxidative stress [34, 35]. To our surprise, RT/TMZ prominently reduced ROS level in intracranial G422^TN^-cells after 3-days of therapy although apoptotic cells were increased (Figs. 4 and 5). Consistent to the ROS decrease, 2-days of RT/TMZ treatment significantly altered GSH metabolism in subcutaneous G422^TN^-tumors (Fig. 1). Since GSH is the major antioxidant, while upregulation of ROS elimination enzymes (ROS_sca_) is a feature of MDR cancer cells, we speculated that ROS_sca_ overexpression contributed to the ROS decrease, thus limited RT/TMZ-induced cytotoxicity and induced G422^TN^-cell MDR. It is well known that PL induces ROS accumulation via depleting GSH in cancer cells [36, 37], so we tested whether PL may surmount MDR of G422^TN^-cells and sensitize RT/TMZ efficacy or not. Indeed, RT/TMZ/PL significantly resumed ROS level and further enhanced apoptosis in G422^TN^-tumor compared to RT/TMZ, while GSH completely reversed PL-synergized effects (Figs. 3, 4 and 5). Although the average ROS level of G422^TN^-cells in RT/TMZ/PL group did not exceed that in PL or control groups, apoptotic cells in RT/TMZ/PL group were the highest among all groups, strongly indicating that the ROS toxic threshold level in RT/TMZ-treated G422^TN^-cells was much lower than that in untreated or PL-treated G422^TN^-cells. Thus, targeting dysregulated ROS decrease by ROS inducer early during RT/TMZ therapy is the key for sensitizing RT/TMZ efficacy in refractory GBM.

PD-1/PD-L1 is the most concerned immune checkpoint, and its upregulation is significantly correlated with the TIM [38], prompting us to further explore the efficacy of αPD1 combined with RT/TMZ/PL therapy. RT/TMZ/PL and RT/TMZ/PL/αPD1 regimens can achieve efficient immune cure in highly refractory preclinical G422^TN^-GBM model, hence deserving a priority for clinical trials. It was estimated that the age of mice after adult was around 1/25 of human being. So the median survival of the untreated and RT/TMZ-treated G422-mice (Figs. 2 and 3) are slightly longer than but very close to that of the untreated and RT/TMZ-treated GBM patients [4]. PL-combined RT/TMZ showed most effective compared to other combinations and achieved LTS (corresponding to 6.9 years in human) in 25% of G422^TN^-mice. It is therefore rational to speculate that the RT/TMZ/PL regimen might greatly enhance the 5-year survival rate of GBM patients, which under current conventional RT/TMZ therapies is merely 9.8% [4]. Importantly, one of the 2 LTS mice passed the rechallenge phase (survived over 100 days), demonstrating RT/TMZ/PL therapy truly cured G422^TN^-GBM. More excitingly, RT/TMZ/PL plus αPD1 doubled the numbers of both LTS mice (50%) and immune-cured mice (25%). While RT/TMZ plus αPD1 could not achieve immune cure (Fig. 7). Clearly, RT/TMZ/PL therapy had remolded the tumor microenvironment, which greatly facilitated αPD1 efficacy in immune-resistant GBM.

PL improved RT/TMZ efficacy in G422^TN^-mice mainly via activating oxidative stress pathways. RT/TMZ/PL-activated oxidative stress damaging makers (i.e., ssGSEA score) significantly correlated to human GBM malignancy and better OS in patients. Consistent to the activation of oxidative stress pathways, PL synergized RT/TMZ to upregulate most of ROS_gen_ and ROS_sca_ signature genes (vs. all other groups, Fig. 4). Only second to RT/TMZ/PL group, RT/TMZ group showed evident overall ROS_gen_ and ROS_sca_ gene upregulation (vs. PL and control) (Fig. 4D). Consistent to our model, RT, TMZ or RT/TMZ therapy upregulated most of ROS_gen_ and ROS_sca_ genes in human LGG and GBM (Fig. 1I). Since ROS level was prominently reduced in RT/TMZ-treated G422^TN^-tumor, ROS is not likely the direct killer of G422^TN^-cells herein. However, oxidative stress pathway or damaging genes were activated or induced by RT/TMZ as most ROS_gen_ and ROS_sca_ genes were upregulated, which may reduce ROS toxic threshold in G422^TN^-cells. Thus, further ROS increase via PL could exacerbate oxidative stress damage in G422^TN^-cells on the basis of RT/TMZ therapy, resulting in a further decrease in proliferation and increase in apoptosis of G422^TN^-cells (Fig. 5A-B). While the toxicity threshold of ROS in PL monotherapy is relatively high, which mainly affect the migration and invasion ability of G422^TN^-cells (Fig. 5A-B). Although alterations of *Gpx2* and *Duox2* expression were most evident in PL or RT/TMZ/PL group, only *SOD2* among all the 27 ROS_gen_/ROS_sca_ genes showed significant correlation to OS of GBM patients (Supplementary Fig. 5). Clearly, activation of oxidative stress in RT/TMZ/PL-treated G422^TN^-cells reflects the collaborating effects of lots of genes in ROS metabolism.

RT/TMZ/PL-activated oxidative stress not only induced apoptosis but also reshaped TIM via inflammation in intracranial G422^TN^-tumors, which greatly contributed to immune cure of G422^TN^-mice. Bulk RNA-seq data clearly showed that RT/TMZ/PL therapy highly upregulated genes in inflammation-associated pathways including cytokine production and NLRP3 inflammation, positively correlating to oxidative stress activation. The prominent upregulation of inflammation pathways in RT/TMZ/PL-treated G422^TN^-tumors was positively correlated to the increase of TAMs, which are the most abundant immune cell types in human GBM [39]. Further, RT/TMZ/PL increased IFN TAMs specifically compared to RT/TMZ, although other TAMs subtypes were also increased compared to PL or control group. IFN TAMs are most likely overlapping with previously M1-like TAMs, as both of them produce similar profiles of pro-inflammatory cytokines such as Stat1, Cxcl9 and Cxcl10 [40, 41], and positively correlate to NLRP3 inflammation pathway activation [42, 43]. It is known that inflammation and TAMs are key players in reshaping TIM. In RT/TMZ/PL-treated G422^TN^-tumors, the simultaneous upregulation of “Antigen presentation” signature genes, CD8^+^ T cell markers and PD-1/PD-L1 genes supported that RT/TMZ/PL-activated inflammation and TAMs had converted the “cold” immune microenvironment to a “hot” one [44, 45] in G422^TN^-tumors. Finally, the “cold” to “hot” immune microenvironment transformation was verified by prominent increase of CD3^+^ and CD8^+^ T-cells in RT/TMZ/PL-treated G422^TN^-tumors. T cells are vital components of tumor microenvironment with different subtypes and grade-dependent spatial heterogeneity in glioma [46]. The increase of CD8^+^ T cells might be the truly therapeutic cells for immune cured G422^TN^-mice in RT/TMZ/PL group. Accompanying CD8^+^ T cell infiltration, the immune suppressive PD1/PL-L1 axis [47] was upregulated. The supplement of αPD-1 abolished the effect of PD1/PL-L1 pair on killing T cells. Meanwhile, we further explored the effect of RT/TMZ/PL therapy on peripheral immunity. Flow cytometry showed that CD4^+^ T cells increased and CD8^+^ T cells decreased in spleen of G422^TN^-mice, the former in line with the changes during therapy [48]. Decrease of CD8^+^ T cells might contribute to its increase into in situ GBM tissue, which were inactivated in the TIM created by RT/TMZ. Surprisingly, PL did not increase the efficacy of RT/TMZ in the periphery, where PL further increased CD4^+^ T cells and decreases CD8^+^ T cells, hinting PL might act primarily in the center. Anyway, there is no doubt that RT/TMZ/PL/αPD1 greatly augmented effect of immune cure in G422^TN^-mice.

## Conclusion

**Fig. 8:**
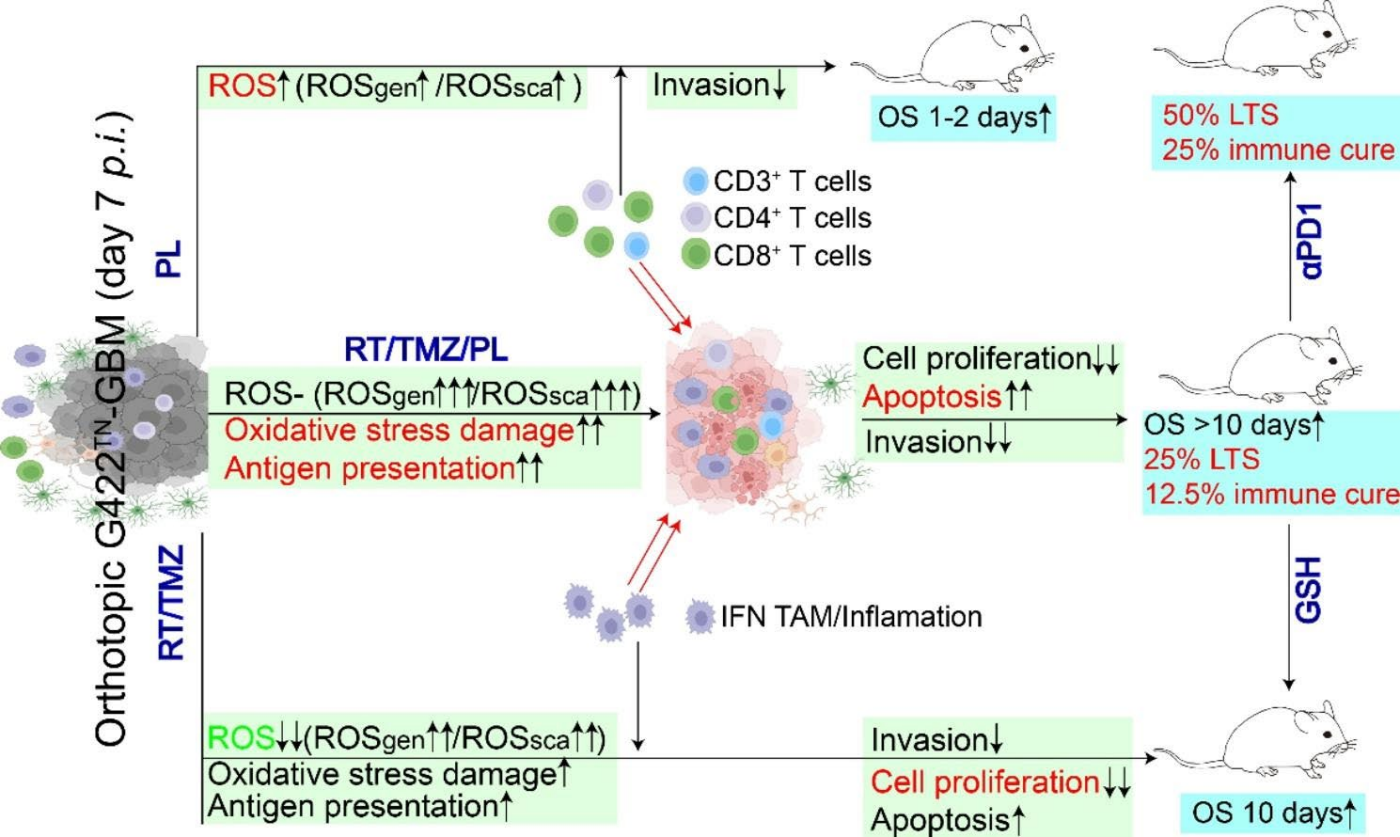
Schematic diagram of the article summary

In summary, the rapid growth of G422^TN^-tumor is accompanied by ROS overproduction, ROS_gen_/ROS_sca_ upregulation and oxidative stress but its cytotoxicity is under control. RT/TMZ exerted its therapeutic effects via suppressing cell proliferation (major) and inducing apoptosis (minor), which was accompanied by TAMs increase/transformation and oxidative stress activation (including ROS_gen_/ROS_sca_ upregulation), reshaping tumor microenvironment toward pro-inflammatory; however, ROS production was greatly reduced as a result of prominent cell proliferation suppression, which restrained oxidative stress damage. PL specifically induced ROS accumulation via inhibiting antioxidant activity and reprograming ROS_gen_/ROS_sca_ expression profile, which slightly improved efficacy. RT/TMZ/PL combinations resumed ROS level by resetting ROS_gen_/ROS_sca_, exacerbated oxidative stress-induced cell death, enhanced inflammatory and immune responses (including IFN TAMs, CD8^+^ T cells), thus converted “cold” tumor microenvironment to “hot” and produced curable efficacy (Fig. 8). αPD-1 blocked RT/TMZ/PL-induced PD-1/PD-L1 inhibitory function, thus RT/TMZ/PL/αPD1 achieved 50% of cure or 25% of immune cure in G422^TN^-mice. These results verified the refractoriness of GBM and strongly suggest clinical trials of RT/TMZ/PL/αPD1 regimen in GBM patients.

## Electronic supplementary material

Below is the link to the electronic supplementary material.

Supplementary Material 1

Supplementary Material 2

Supplementary Material 3

Supplementary Material 4

Supplementary Material 5

Supplementary Material 6

Supplementary Material 7

Supplementary Material 8

## Data Availability

Data are available for reasonable request. Raw data were uploaded in Gene Expression Omnibus (GEO) database with BioProject ID PRJNA821400, and integrated data were uploaded as supplementary information.

## Abbreviations

αPD1: anti-PD-1 antibody immunotherapy
BLI: bioluminescent imaging
DHE: dihydroethidium
DM: differential metabolite
G422^TN−^GBM: triple-negative G422-GBM
GBM: glioblastoma multiforme
GSH: glutathione
LGG: lower grade glioma
LTS: long-term survival
MDR: multidrug resistance
*p.i.*: post implantation
Pl: piperlongumine
OS: overall survival
ROS: reactive oxygen species
ROSgen: ROS generation signature genes
ROSsca: ROS scavenging signature genes
RT/TMZ: temozolomide concurrent radiotherapy
ssGSEA: single-sample GSEA
TAM: tumor-associated macrophage
TIM: tumor immunosuppressive microenvironment
WBI: whole brain irradiation

## References

[1] Louis DN, Perry A, Reifenberger G, von Deimling A, Figarella-Branger D, Cavenee WK (2016). The 2016 World Health Organization classification of tumors of the Central Nervous System: a summary. Acta Neuropathol.

[2] Weller M, Wick W, Aldape K, Brada M, Berger M, Pfister SM (2015). Glioma. Nat Rev Dis Primers.

[3] Stupp R, Mason WP, van den Bent MJ, Weller M, Fisher B, Taphoorn MJ (2005). Radiotherapy plus concomitant and adjuvant temozolomide for glioblastoma. N Engl J Med.

[4] Stupp R, Hegi ME, Mason WP, van den Bent MJ, Taphoorn MJ, Janzer RC (2009). Effects of radiotherapy with concomitant and adjuvant temozolomide versus radiotherapy alone on survival in glioblastoma in a randomised phase III study: 5-year analysis of the EORTC-NCIC trial. Lancet Oncol.

[5] Yang F, He Z, Duan H, Zhang D, Li J, Yang H (2021). Synergistic immunotherapy of glioblastoma by dual targeting of IL-6 and CD40. Nat Commun.

[6] Zheng Z, Zhang J, Jiang J, He Y, Zhang W, Mo X, et al. Remodeling tumor immune microenvironment (TIME) for glioma therapy using multi-targeting liposomal codelivery. J Immunother Cancer. 2020;8(2). 10.1136/jitc-2019-000207 PMC7437977 32817393

[7] Qiu L, Meng Y, Han J (2022). STING cg16983159 methylation: a key factor for glioblastoma immunosuppression. Signal Transduct Target Ther.

[8] Panagioti E, Kurokawa C, Viker K, Ammayappan A, Anderson SK, Sotiriou S, et al. Immunostimulatory bacterial antigen-armed oncolytic measles virotherapy significantly increases the potency of anti-PD1 checkpoint therapy. J Clin Invest. 2021;131(13). 10.1172/JCI141614 PMC8245183 34196308

[9] Senft C, Bink A, Franz K, Vatter H, Gasser T, Seifert V (2011). Intraoperative MRI guidance and extent of resection in glioma surgery: a randomised, controlled trial. Lancet Oncol.

[10] Yamada S, Muragaki Y, Maruyama T, Komori T, Okada Y (2015). Role of neurochemical navigation with 5-aminolevulinic acid during intraoperative MRI-guided resection of intracranial malignant gliomas. Clin Neurol Neurosurg.

[11] Cui Q, Wang JQ, Assaraf YG, Ren L, Gupta P, Wei L (2018). Modulating ROS to overcome multidrug resistance in cancer. Drug Resist Updat.

[12] Guishard AF, Yakisich JS, Azad N, Iyer AKV (2018). Translational gap in ongoing clinical trials for glioma. J Clin Neurosci.

[13] Liu F, Xu X, Li C, Li C, Li Y, Yin S (2020). Mannose synergizes with chemoradiotherapy to cure cancer via metabolically targeting HIF-1 in a novel triple-negative glioblastoma mouse model. Clin Transl Med.

[14] Liu F, Xu XH, Li CY, Zhang TT, Yin SL, Liu GQ (2021). Rapid tumor recurrence in a novel murine GBM surgical model is associated with Akt/PD-L1/vimentin signaling. Biochem Biophys Res Commun.

[15] Lei K, Gu X, Alvarado AG, Du Y, Luo S, Ahn EH (2020). Discovery of a dual inhibitor of NQO1 and GSTP1 for treating glioblastoma. J Hematol Oncol.

[16] Hayes JD, Dinkova-Kostova AT, Tew KD (2020). Oxidative stress in Cancer. Cancer Cell.

[17] Zheng L, Fang S, Chen A, Chen W, Qiao E, Chen M (2022). Piperlongumine synergistically enhances the antitumour activity of sorafenib by mediating ROS-AMPK activation and targeting CPSF7 in liver cancer. Pharmacol Res.

[18] Qiu XY, Hu DX, Chen WQ, Chen RQ, Qian SR, Li CY (2018). PD-L1 confers glioblastoma multiforme malignancy via ras binding and Ras/Erk/EMT activation. Biochim Biophys Acta Mol Basis Dis.

[19] Dobin A, Davis CA, Schlesinger F, Drenkow J, Zaleski C, Jha S (2013). STAR: ultrafast universal RNA-seq aligner. Bioinformatics.

[20] Li B, Dewey CN (2011). RSEM: accurate transcript quantification from RNA-Seq data with or without a reference genome. BMC Bioinformatics.

[21] Love MI, Huber W, Anders S (2014). Moderated estimation of fold change and dispersion for RNA-seq data with DESeq2. Genome Biol.

[22] Kanehisa M, Furumichi M, Tanabe M, Sato Y, Morishima K (2017). KEGG: new perspectives on genomes, pathways, diseases and drugs. Nucleic Acids Res.

[23] Yu G, Wang LG, Han Y, He QY (2012). clusterProfiler: an R package for comparing biological themes among gene clusters. Omics.

[24] Barbie DA, Tamayo P, Boehm JS, Kim SY, Moody SE, Dunn IF (2009). Systematic RNA interference reveals that oncogenic KRAS-driven cancers require TBK1. Nature.

[25] Becht E, de Reyniès A, Giraldo NA, Pilati C, Buttard B, Lacroix L (2016). Immune and Stromal classification of Colorectal Cancer is Associated with Molecular Subtypes and relevant for Precision Immunotherapy. Clin Cancer Res.

[26] Niu B, Liao K, Zhou Y, Wen T, Quan G, Pan X (2021). Application of glutathione depletion in cancer therapy: enhanced ROS-based therapy, ferroptosis, and chemotherapy. Biomaterials.

[27] Tripathi SK, Biswal BK (2020). Piperlongumine, a potent anticancer phytotherapeutic: perspectives on contemporary status and future possibilities as an anticancer agent. Pharmacol Res.

[28] Khalsa JK, Cheng N, Keegan J, Chaudry A, Driver J, Bi WL (2020). Immune phenotyping of diverse syngeneic murine brain tumors identifies immunologically distinct types. Nat Commun.

[29] Fan D, Yue Q, Chen J, Wang C, Yu R, Jin Z (2021). Reprogramming the immunosuppressive microenvironment of IDH1 wild-type glioblastoma by blocking wnt signaling between microglia and cancer cells. Oncoimmunology.

[30] Pombo Antunes AR, Scheyltjens I, Lodi F, Messiaen J, Antoranz A, Duerinck J (2021). Single-cell profiling of myeloid cells in glioblastoma across species and disease stage reveals macrophage competition and specialization. Nat Neurosci.

[31] Kim SS, Harford JB, Moghe M, Slaughter T, Doherty C, Chang EH (2019). A tumor-targeting nanomedicine carrying the p53 gene crosses the blood-brain barrier and enhances anti-PD-1 immunotherapy in mouse models of glioblastoma. Int J Cancer.

[32] Lei K, Kang SS, Ahn EH, Chen C, Liao J, Liu X (2021). C/EBPβ/AEP Signaling regulates the oxidative stress in malignant cancers, stimulating the Metastasis. Mol Cancer Ther.

[33] Liu QR, Liu JM, Chen Y, Xie XQ, Xiong XX, Qiu XY (2014). Piperlongumine inhibits migration of glioblastoma cells via activation of ROS-dependent p38 and JNK signaling pathways. Oxid Med Cell Longev.

[34] He C, Lu S, Wang XZ, Wang CC, Wang L, Liang SP (2021). FOXO3a protects glioma cells against temozolomide-induced DNA double strand breaks via promotion of BNIP3-mediated mitophagy. Acta Pharmacol Sin.

[35] Tabatabaie F, Franich R, Feltis B, Geso M. Oxidative damage to Mitochondria enhanced by Ionising Radiation and Gold Nanoparticles in Cancer cells. Int J Mol Sci. 2022;23(13). 10.3390/ijms23136887 PMC9266628 35805905

[36] Li L, Zhao Y, Cao R, Li L, Cai G, Li J (2019). Activity-based protein profiling reveals GSTO1 as the covalent target of piperlongumine and a promising target for combination therapy for cancer. Chem Commun (Camb).

[37] Wang H, Jiang H, Corbet C, de Mey S, Law K, Gevaert T (2019). Piperlongumine increases sensitivity of colorectal cancer cells to radiation: involvement of ROS production via dual inhibition of glutathione and thioredoxin systems. Cancer Lett.

[38] Escors D, Gato-Cañas M, Zuazo M, Arasanz H, García-Granda MJ, Vera R (2018). The intracellular signalosome of PD-L1 in cancer cells. Signal Transduct Target Ther.

[39] Dumas AA, Pomella N, Rosser G, Guglielmi L, Vinel C, Millner TO (2020). Microglia promote glioblastoma via mTOR-mediated immunosuppression of the tumour microenvironment. Embo j.

[40] Wu K, Yuan Y, Yu H, Dai X, Wang S, Sun Z (2020). The gut microbial metabolite trimethylamine N-oxide aggravates GVHD by inducing M1 macrophage polarization in mice. Blood.

[41] Lu G, Zhang R, Geng S, Peng L, Jayaraman P, Chen C (2015). Myeloid cell-derived inducible nitric oxide synthase suppresses M1 macrophage polarization. Nat Commun.

[42] Jourdan T, Godlewski G, Cinar R, Bertola A, Szanda G, Liu J (2013). Activation of the Nlrp3 inflammasome in infiltrating macrophages by endocannabinoids mediates beta cell loss in type 2 diabetes. Nat Med.

[43] Lv LL, Feng Y, Wu M, Wang B, Li ZL, Zhong X (2020). Exosomal miRNA-19b-3p of tubular epithelial cells promotes M1 macrophage activation in kidney injury. Cell Death Differ.

[44] Weiss T, Puca E, Silginer M, Hemmerle T, Pazahr S, Bink A, et al. Immunocytokines are a promising immunotherapeutic approach against glioblastoma. Sci Transl Med. 2020;12(564). 10.1126/scitranslmed.abb2311 33028706

[45] Vom Berg J, Vrohlings M, Haller S, Haimovici A, Kulig P, Sledzinska A (2013). Intratumoral IL-12 combined with CTLA-4 blockade elicits T cell-mediated glioma rejection. J Exp Med.

[46] Robinson MH, Vasquez J, Kaushal A, MacDonald TJ, Velázquez Vega JE, Schniederjan M, et al. Subtype and grade-dependent spatial heterogeneity of T-cell infiltration in pediatric glioma. J Immunother Cancer. 2020;8(2). 10.1136/jitc-2020-001066 PMC7422651 32788236

[47] Reardon DA, Brandes AA, Omuro A, Mulholland P, Lim M, Wick A (2020). Effect of Nivolumab vs Bevacizumab in patients with recurrent glioblastoma: the CheckMate 143 phase 3 Randomized Clinical Trial. JAMA Oncol.

[48] Fecci PE, Ochiai H, Mitchell DA, Grossi PM, Sweeney AE, Archer GE (2007). Systemic CTLA-4 blockade ameliorates glioma-induced changes to the CD4 + T cell compartment without affecting regulatory T-cell function. Clin Cancer Res.

