# Supplementary material for: Piperlongumine conquers temozolomide chemoradiotherapy resistance to achieve immune cure in refractory glioblastoma via boosting oxidative stress-inflamation-CD8+-T cell immunity
Source: J Exp Clin Cancer Res. 2023 May 10;42:118. doi: 10.1186/s13046-023-02686-1 (PMC10170830; doi:10.1186/s13046-023-02686-1)
Supplement: Supplementary file 2 — Supplementary Material 2 [file 13046_2023_2686_MOESM2_ESM.docx]

**Piperlongumine synergizes temozolomide chemoradiotherapy to achieve immune cure in refractory glioblastoma via boosting CD8^+^-T cell immunity**

Feng Liu^1,2,†^, Qian Zhou^3,†^, Hai-feng Jiang^1,†^, Ting-ting Zhang^1^, Cheng Miao^1^, Xiao-hong Xu^1^, Jia-xing Wu^1^, Song-lin Yin^1^, Shi-jie Xu^1^, Jing-yi Peng^1^, Pan-pan Gao^1^, Xuan Cao^4,*^, Feng Pan^5,*^, Ximiao He^3,*^, Xiao Qian Chen^1,*^

**Supplementary Materials and Methods**

**Experiment of the blind stochastic principle and inclusion and exclusion criteria**

All orthotopic G422^TN^-mouse models were established by Feng Liu and randomized. All mice were subjected to *in vivo* optical imaging, and the mice with no signal in the head were excluded, and the remaining mice were randomly grouped according to the optical density value (ROI). The treatment was performed by Hai-feng Jiang, and the evaluation of treatment effect (OS, body weight, tumor volume) was performed by Ting-ting Zhang, Cheng Miao, Xiao-hong Xu, and Jia-xing Wu, respectively. Professor Chen mastered all blind random assignment.

**Western blotting analysis**

Adult Kunming male mice bearing subcutaneous G422^TN^-tumors of 0.8-1 cm in diameter were treated with vehicle (control), PL (5 mg/kg/d), RT/TMZ (total body irradiation, 10 Gy; plus TMZ 50 mg/kg/d) or RT/TMZ/PL (RT/TMZ plus PL) for 2 days and then the subcutaneous tumor tissues were isolated for Western blotting analyses (n=3/group). Briefly, G422 tumor mass tissue cell lysates were collected and dispersed in radioimmunoprecipitation assay lysis buffer containing phenylmethanesulfonyl fluoride. Equal amounts of total proteins were subjected to sodium dodecyl sulfate polyacrylamide gel electrophoresis and electrotransferred onto nitrocellulose filter membranes (NC, 0.45 μm, Merck Millipore, Cork, Ireland). The membranes were incubated with specific primary antibodies: anti-B2M (1:1000, E-AB-40355, Elabscience, China), anti-IL6 (1:1000, WL02841, Wanleibio, China), anti-SOD1 (1:1000, WL01846, Wanleibio, China) and anti-β-actin (1:1500, GB15003, Servicebio, China). The horseradish peroxidase-conjugated secondary antibody was used for blotting. Following antibody incubation, intensity of each protein band on the NC membrane was visualized and quantified using the enhanced chemiluminescence system (BIO-RAD, USA).

**Supplementary figures and figure legends**


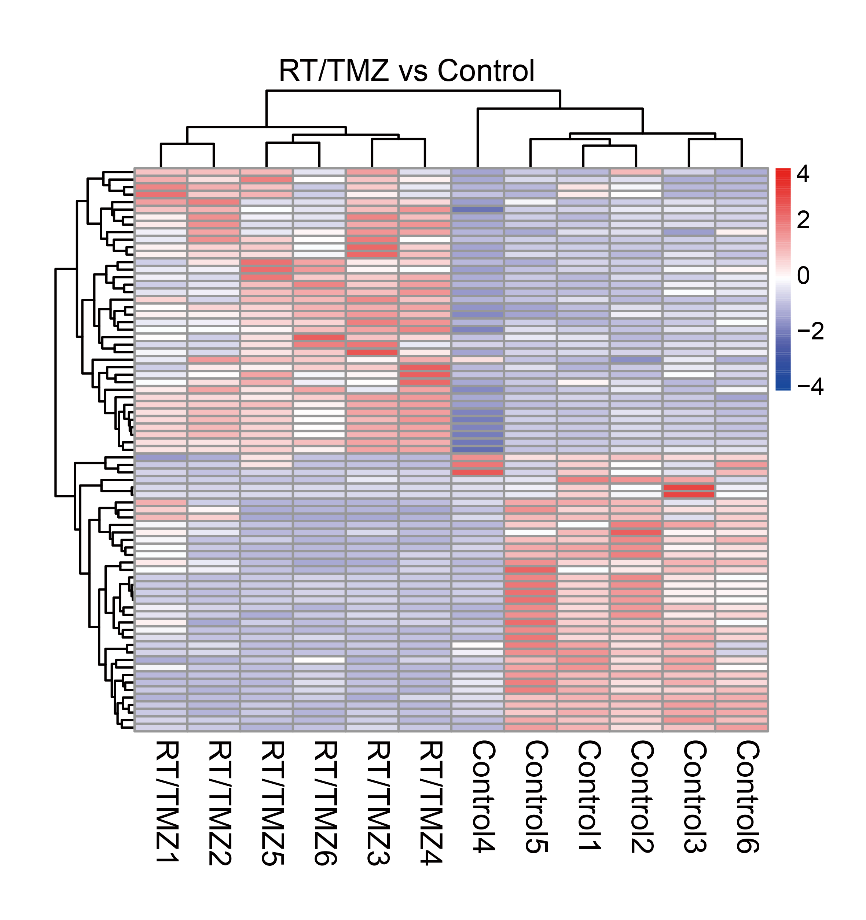


**Supplementary Fig. 1** The heatmap of all metabolites between RT/TMZ and control groups.


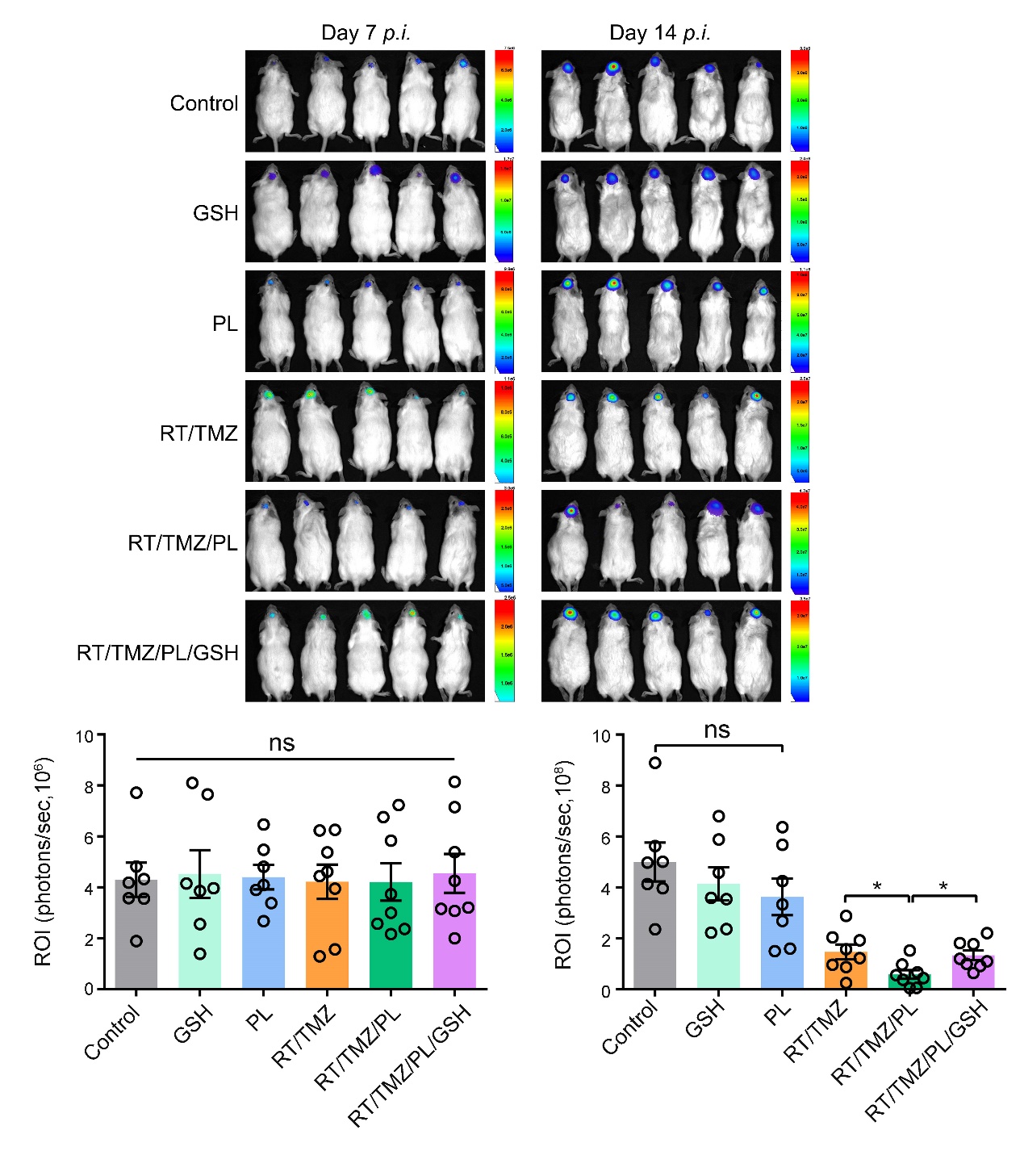


**Supplementary Fig. 2** PL improves RT/TMZ efficacy in G422^TN^-mice. Representative bioluminescent images (upper) and statistical analysis of the ROI values (below) of the intracranial G422^TN^-tumors in control, GSH, PL, RT/TMZ, RT/TMZ/PL and RT/TMZ/PL/GSH group monitored on day 7 and 14. (n=7-8 /group). (Student’s t-tests, **P* < 0.05)


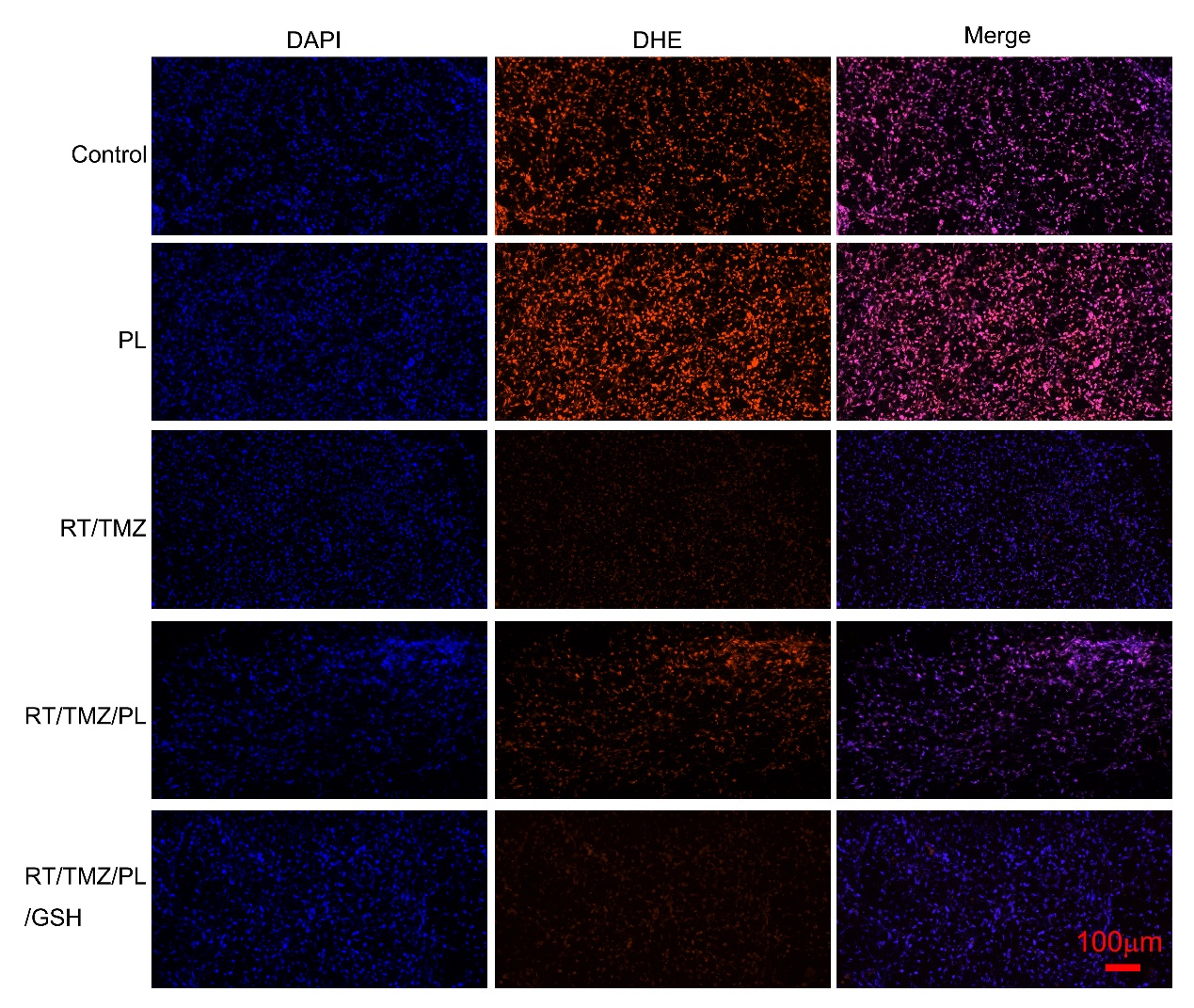


**Supplementary Fig. 3** Effects of PL on ROS levels in different groups. Representative DHE staining of ROS levels in control, PL, RT/TMZ, RT/TMZ/PL, RT/TMZ/PL/GSH group of G422^TN^-mice. Scale bar, 100 μm. (n=3)


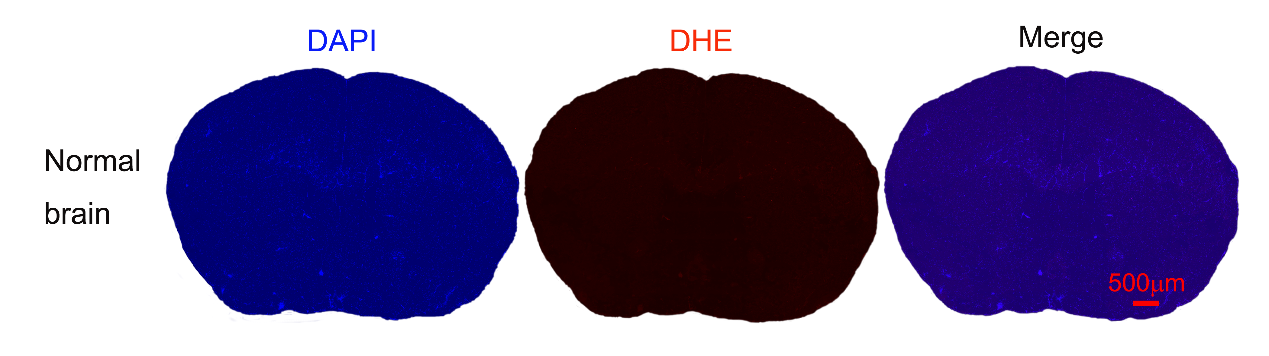


**Supplementary Fig. 4** Normal mice have very low ROS levels in their brains. Scale bar, 500 μm. (n=1).


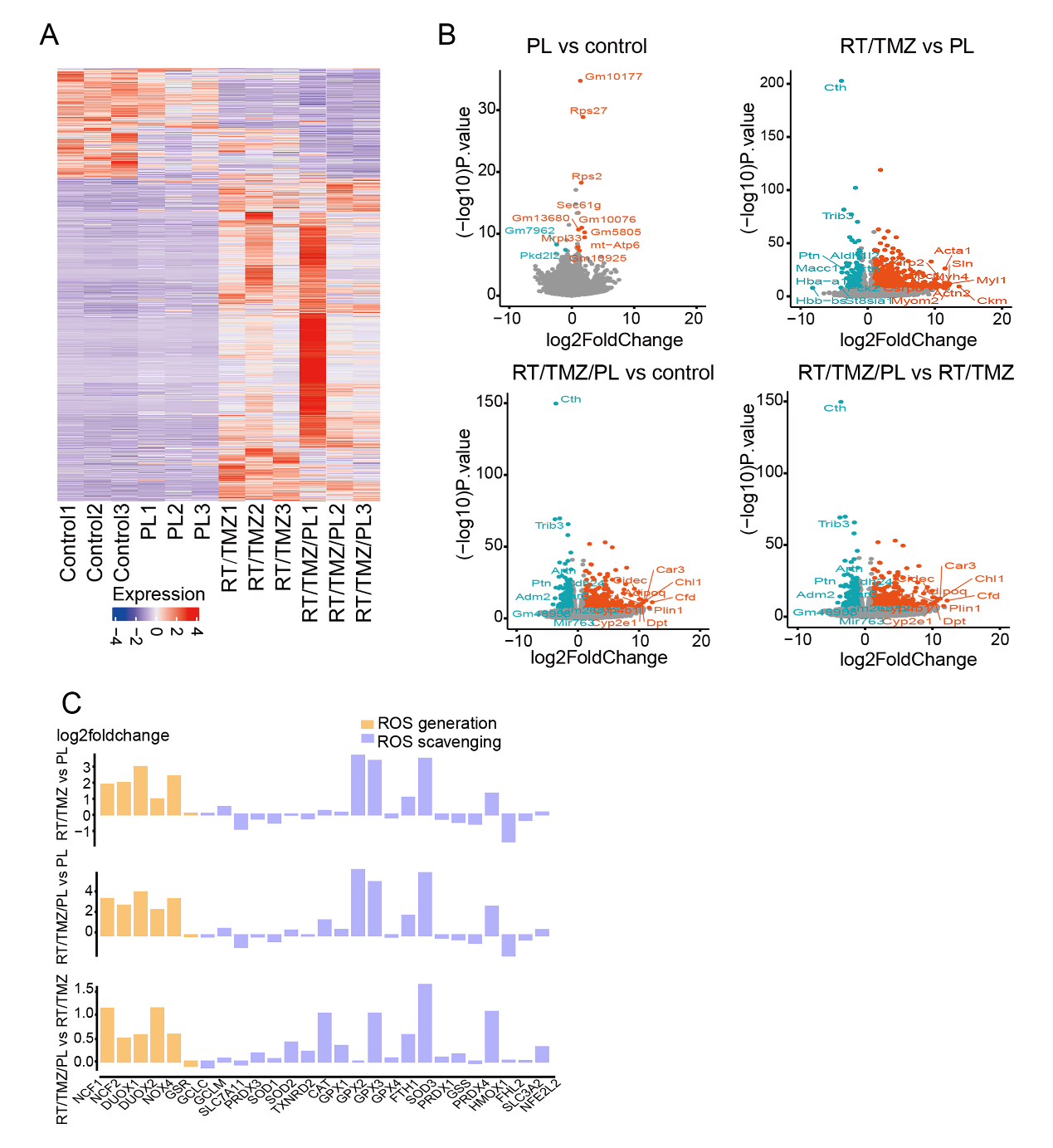
 **Supplementary Fig. 5** Analysis of DEGs in different groups. **A** The heatmap of all DEGs in all groups. **B** Volcanic plot of DEGs between PL and control groups, between RT/TMZ and control groups, between RT/TMZ/PL and control groups, or between RT/TMZ/PL and RT/TMZ groups. **C** The fold changes of gene expression of ROS generating and scavenging genes in different comparison (RT/TMZ treatment vs. PL, top), (RT/TMZ/PL treatment vs. PL, middle), and (RT/TMZ/PL treatment vs. RT/TMZ, bottom).


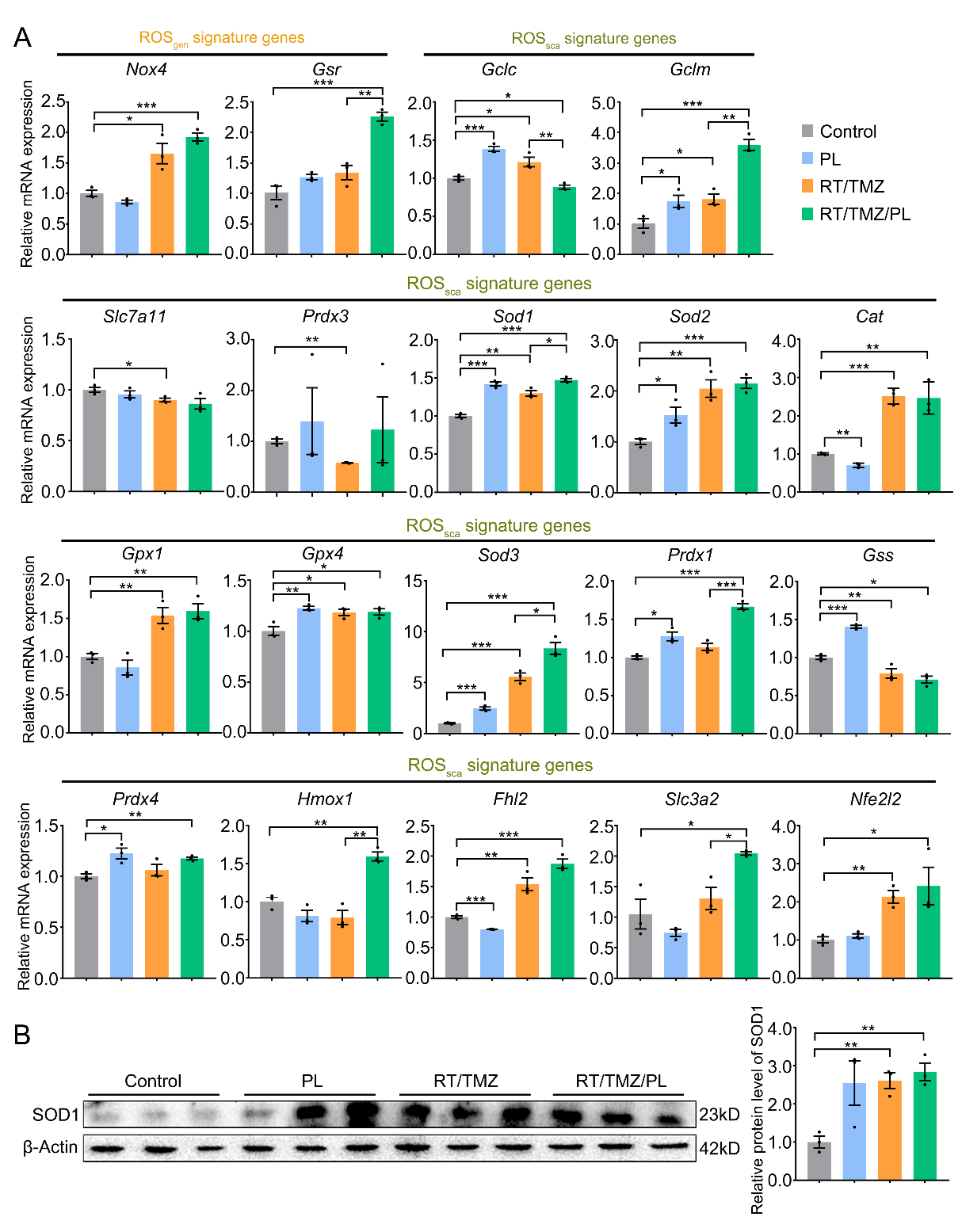


**Supplementary Fig. 6** Verification of ROS relevant DEGs in control, PL, RT/TMZ, RT/TMZ/PL group. **A** qRT-PCR showing the mRNA expression of ROS_gen_ (*Nox4, Gsr*) and ROS_sca_ signature genes (*Gclc, Gclm, Slc7a11, Prdx3, Sod1, Sod2, Cat, Gpx1, Gpx4, Sod3, Prdx1, Gss, Prdx4, Hmox1, Fhl2, Slc3a2, Nfe2l2*). (n=3). **B** Representative graphs (left panel) and statistical analysis (right panel) of the expression of SOD1 in different groups by Western blot. (n=3). (**P* < 0.05, ***P* < 0.01, ****P* < 0.001)


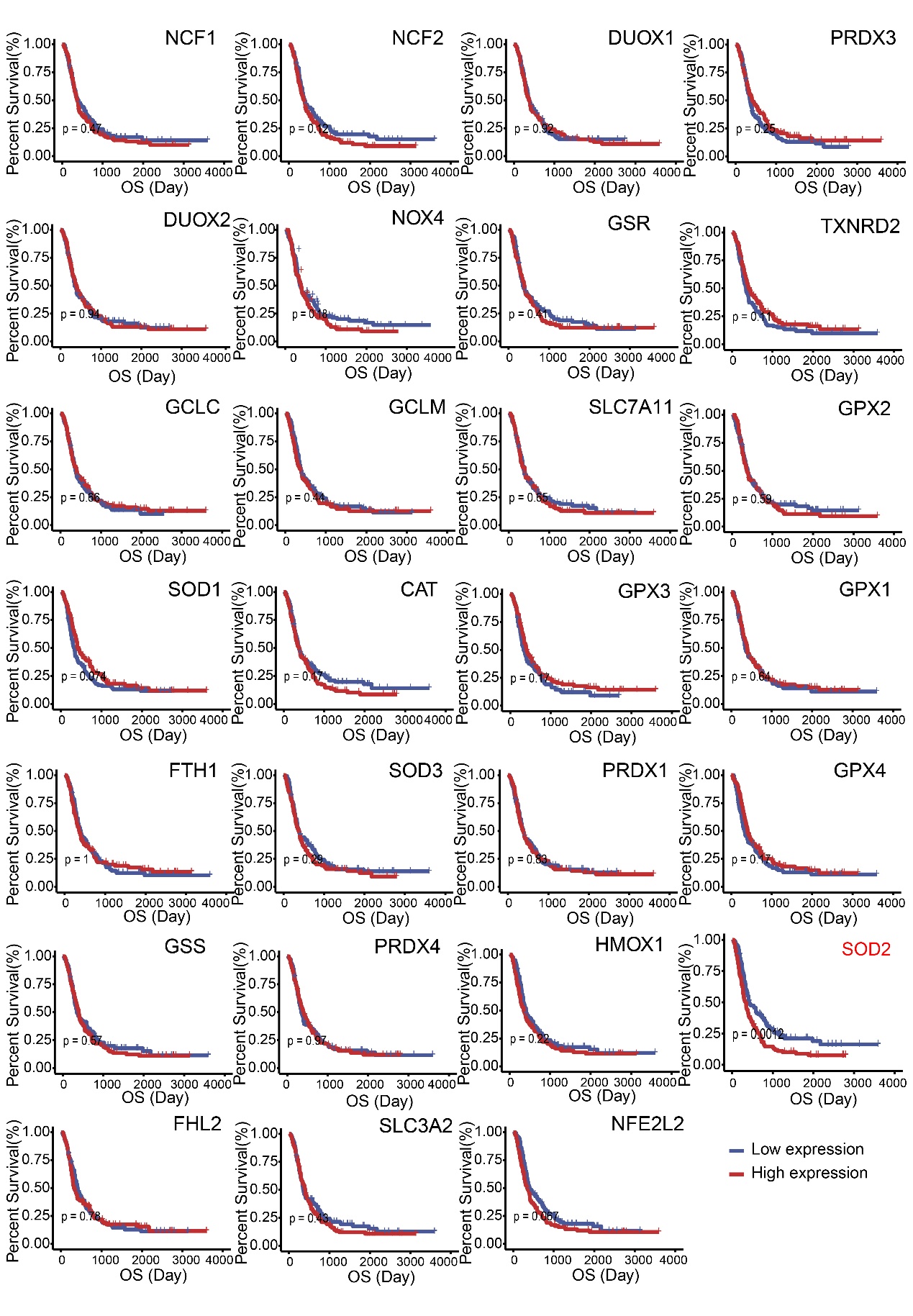


**Supplementary Fig. 7** Relationship between GBM patient survival and 21 ROS signature genes at different level in CGGA GBM dataset.


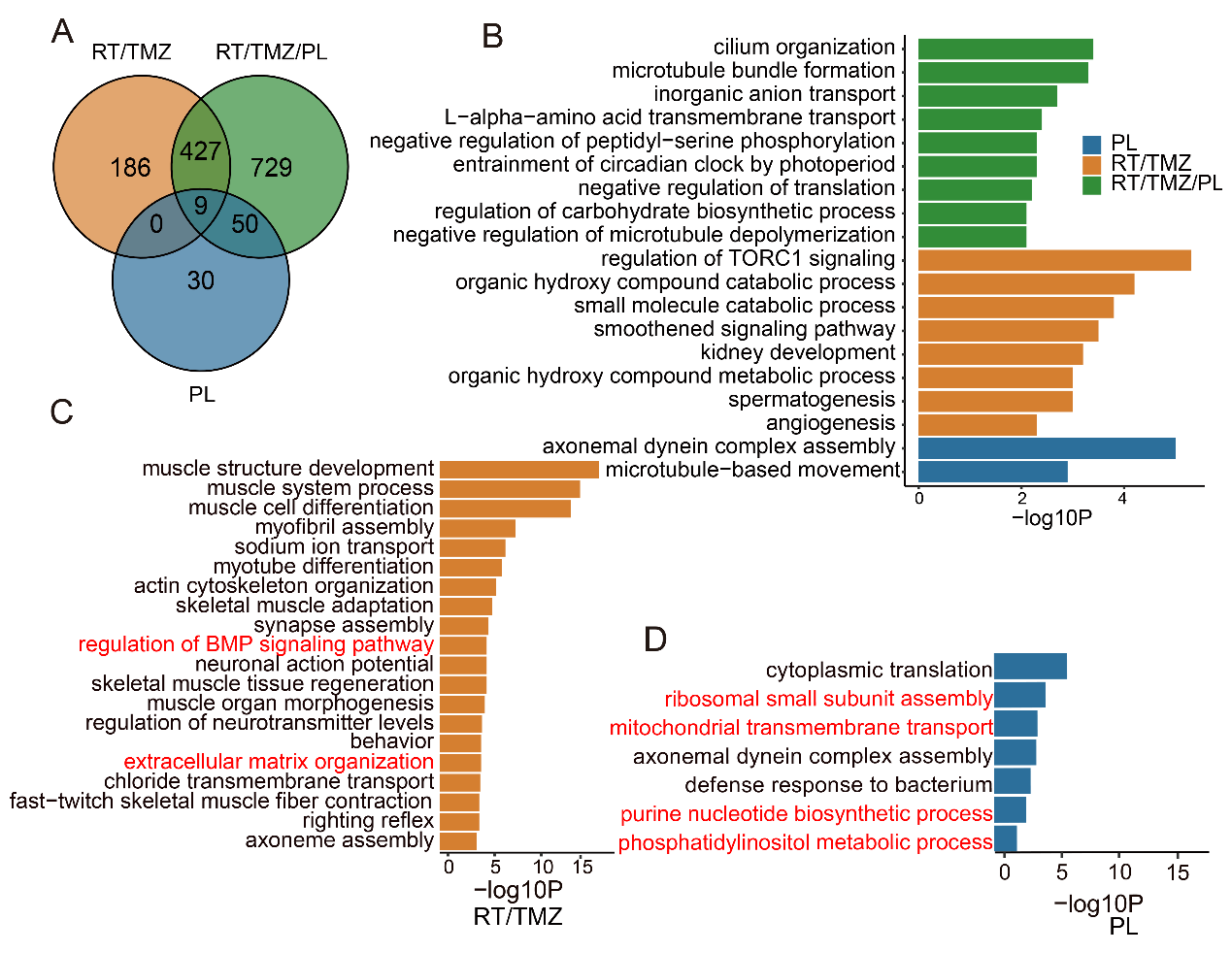


**Supplementary Fig. 8** Venn plot and KEGG analysis of downregulated or upregulated genes in different groups. **A** Venn plot of downregulated genes. **B** KEGG analysis of downregulated genes only present in PL treatment vs control, RT/TMZ treatment vs control, and RT/TMZ/PL vs control, respectively. **C** KEGG analysis of upregulated genes only present in RT/TMZ treatment vs control. **D** KEGG analysis of upregulated genes only present in PL treatment vs control.


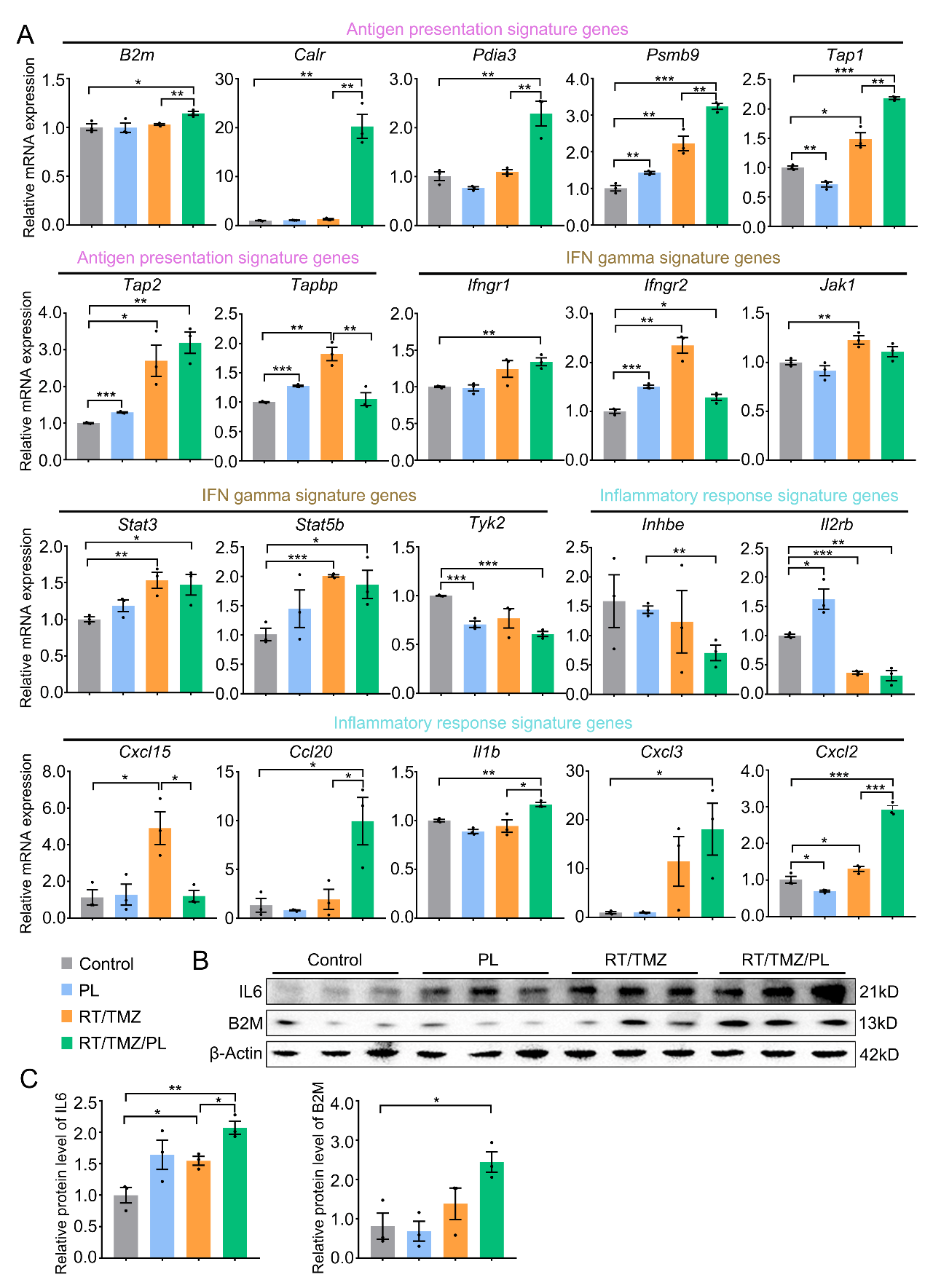


**Supplementary Fig. 9** Verification of the antigen presentation signature genes, the IFN gamma signature genes, and the inflammatory responses signature genes in control, PL, RT/TMZ, RT/TMZ/PL group. **A** qRT-PCR showing the mRNA expression of the antigen presentation signature genes (*B2m, Calr, Pdia3, Psmb9, Tap1, Tap2, Tapbp*), the IFN gamma signature genes (*Ifngr1, Ifngr2, Jak1, Stat3, Stat5b, Tyk2*) and the inflammatory response signature genes (*Inhbe, Il2rb, Cxcl15, Ccl20, Il1b, Cxcl3, Cxcl2*). (n=3). **B** Representative graphs (left panel) and statistical analysis (right panel) of the expression of IL6 and B2M in different groups by Western blot. (n=3). (**P* < 0.05, ***P* < 0.01, ****P* < 0.001)


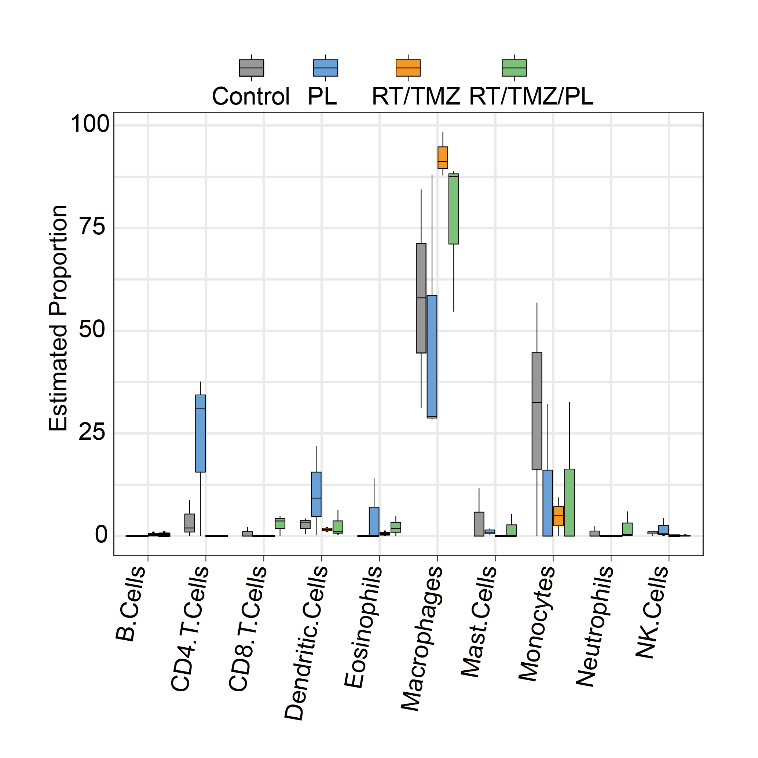


**Supplementary Fig. 10** Evaluation of immune infiltration in different groups.


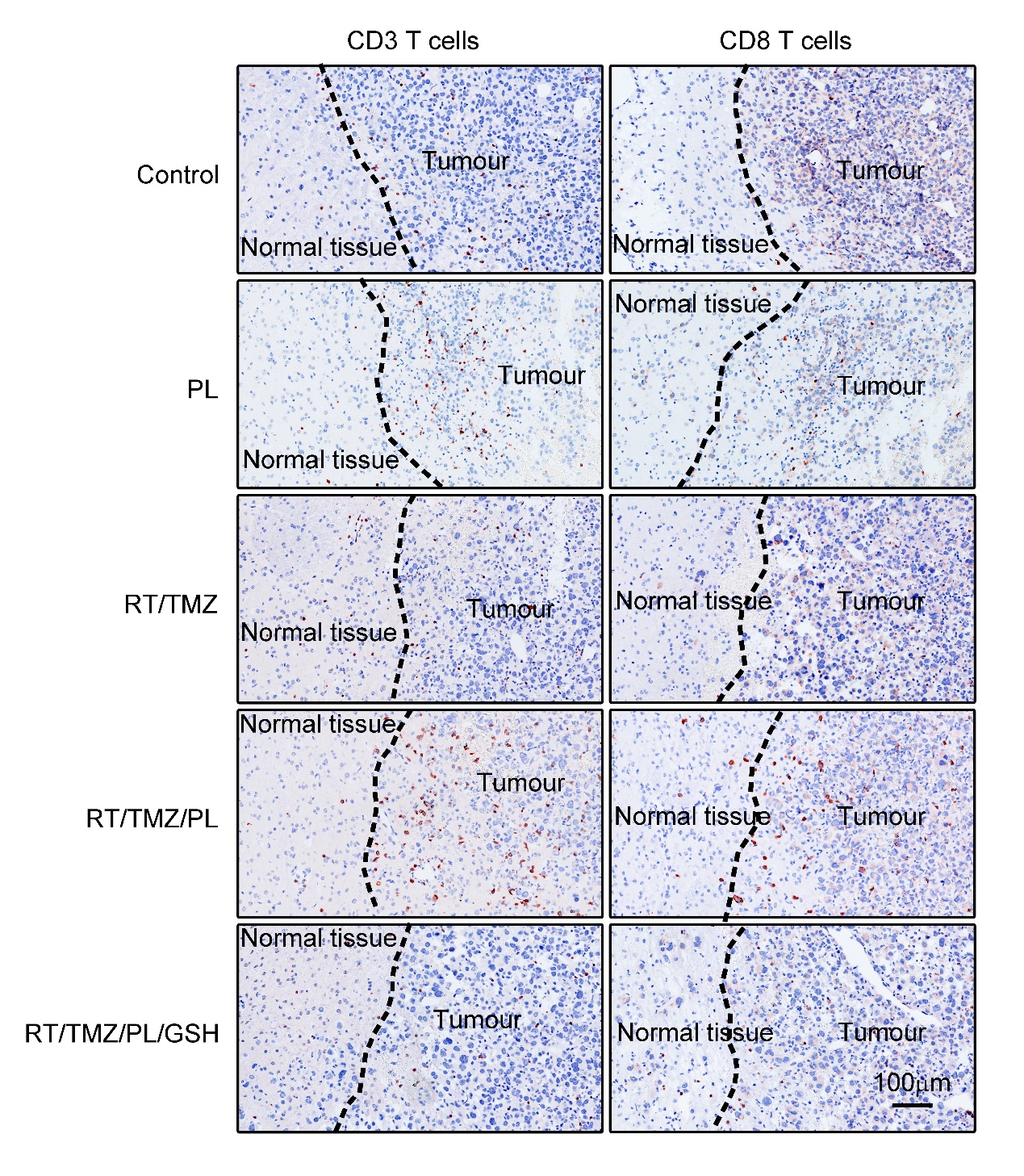


**Supplementary Fig. 11** Effect of different treatments on T cell infiltration into tumor and normal tissues in G422^TN^-mice. IHC staining (CD3 and CD8) of G422^TN^-tumor in control, PL, RT/TMZ, RT/TMZ/PL, RT/TMZ/PL/GSH group on day 9 *p.i.* Scale bar, 100 μm. (n=6)


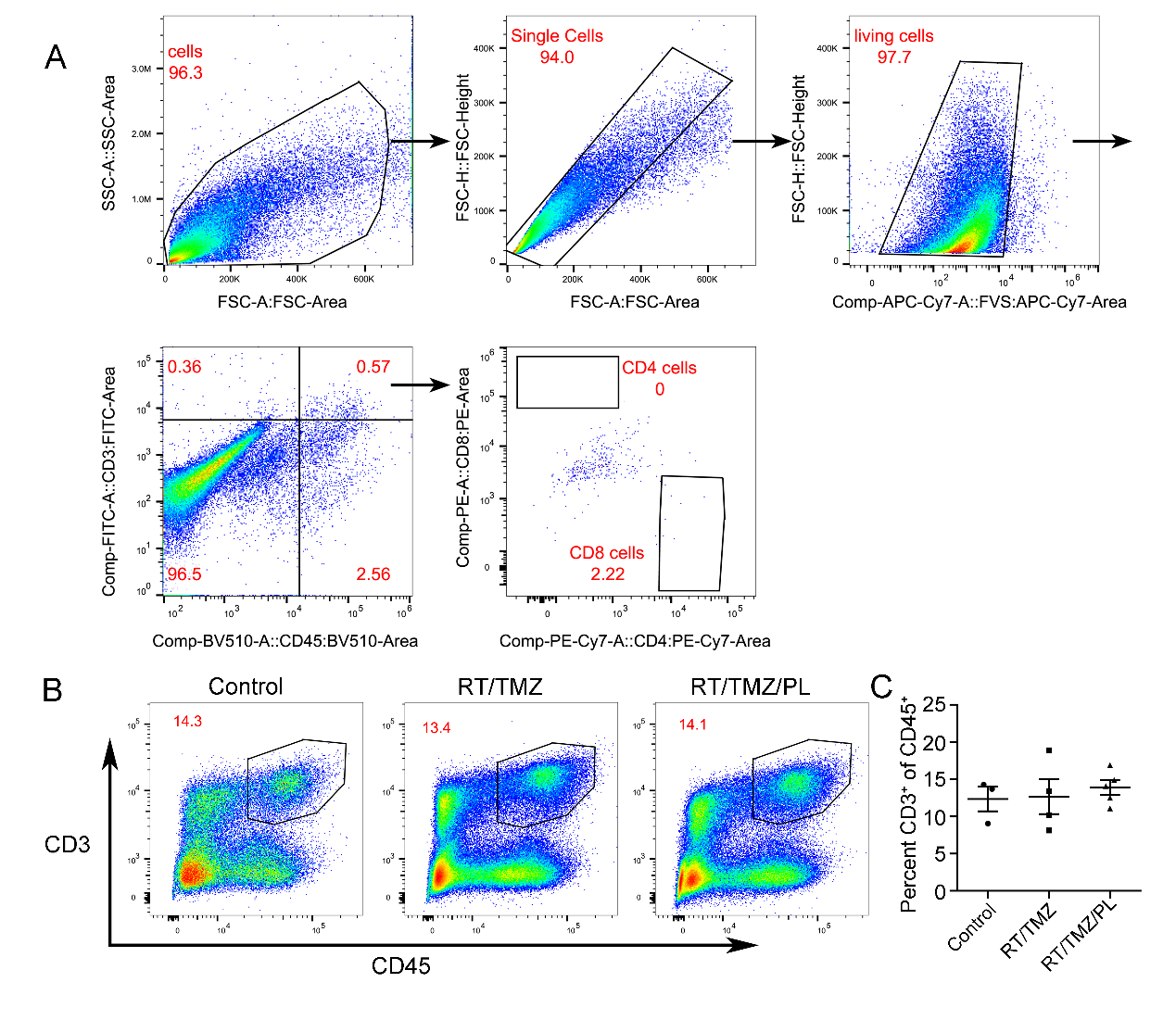


**Supplementary Fig. 12** Effect of different treatments on T cell of brains and spleens in G422^TN^-mice. **A** T cell gating strategy was detected by flow cytometry in different groups of mouse brain (i.e., RT/TMZ/PL group). **B and C** Flow cytometric analysis of T cell gating and statistical analysis in spleen of mice of different groups (control, RT/TMZ and RT/TMZ/PL, n=3-5)

**Supplementary Tables**

**Supplementary table 1.** All differential metabolites of control and RT/TMZ groups.

**Supplementary table 2.** Primer sequences of target genes and reference gene.

**Supplementary table 3.** The quantified Gene expressions of control, PL, RT/TMZ and RT/TMZ/PL groups.

**Supplementary table 4.** The define standards to DEGs of control, PL, RT/TMZ and RT/TMZ/PL groups.

**Supplementary table 5.** The signature genes of hypoxic, IFN, lipid and transitory TAMs.

**Supplementary table 6.** The ROS signature genes of GBM patients in CGGA database.

**Supplementary table 7.** The oxidative stress positive genes and the top 50 upregulated DEGs in RT/TMZ/PL group.
